# A comprehensive overview of recent progress in MXene-based polymer composites: Their fabrication processes, advanced applications, and prospects

**DOI:** 10.1016/j.heliyon.2024.e37030

**Published:** 2024-08-30

**Authors:** Md Injamamul Haque Protyai, Adib Bin Rashid

**Affiliations:** a Department of Mechanical and Production Engineering, Ahsanullah University of Science and Technology, Dhaka, Bangladesh; b Department of Mechanical Engineering, Military Institute of Science and Technology, Dhaka, Bangladesh

**Keywords:** MXenes, Nanofillers, Polymer composites, Protective coatings, EMI shielding

## Abstract

MXenes are a group of 2D transition metal carbonitrides, nitrides and carbides that have become widely recognized as useful materials since they were first discovered in 2011. MXenes, with their exceptional layered structures and splendid external chemistries, have excellent electrical, optical, and thermal properties, making them suitable for catalysis, biomedical uses, environmental remediation, energy storage, and EMI shielding. Over forty MXene compounds with surface terminations like hydroxyl, oxygen, or fluorine are hydrophilic and easily integrated into various applications. Advanced synthesis methods, including selective etching and etchant modifications, have broadened MXene surface chemistries for customized mechanical, thermal, and electrical applications. Integrating MXenes into polymer composites has demonstrated notable promise, enhancing the host polymers' electrical conductivity, thermal stability and mechanical strength. The MXene-polymer composites demonstrate remarkable prospective on behalf of advanced purposes, including flexible electronics, high-performance EMI shielding materials, and lightweight structural components. MXenes have the desirable characteristic of being able to create flexible and translucent films, as well as improve the properties of polymer matrices. This makes them very suitable for use in advanced technological applications. This review summarizes MXene research, methods, and insights, highlighting key discoveries and future directions. This also highlights the importance of ongoing research to fill in the gaps in current knowledge and improve the practical uses of MXenes.

## Graphical abstract

**Figure:**
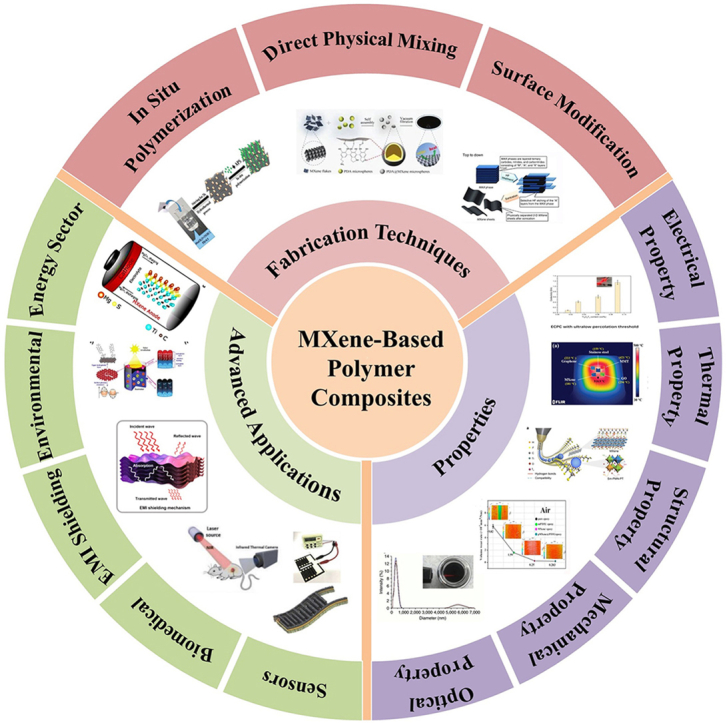

## 1 Introduction

Extensive research has been conducted on two-dimensional (2D) materials in the last 15 years, primarily focusing on the distinctive physical properties of single-layer graphene. This curiosity sparked a surge in scientific investigation into widely recognized 2D materials such as metal boron nitride and dichalcogenides and the identification of numerous novel 2D materials. Certain materials have gained attention for their appealing characteristics and have been applied in practical applications, while others are mostly studied in academic settings. The rapidly expanding two-dimensional materials group includes the transition metal nitrides and carbides, also known as MXenes. MXene is a collection of two-dimensional (2D) materials made of transition metal carbides, nitrides, or carbonitrides. In 2011, the entities were found by researchers at Drexel University in Philadelphia [1]. The word “MXene” is derived from its composition: “M" represents a transition metal, “X" represents carbon or nitrogen, and the suffix “ene” emphasizes its two-dimensional character. MXenes are commonly synthesized through the targeted removal of aluminum layers from layered ternary nitrides or carbides, referred to as MAX phases [2]. Selective etching is a method employed to produce a stratified arrangement comprising transition metal carbides or nitrides, commonly referred to as MXenes. The MXene layers that are formed resemble graphene in their two-dimensional structure, but they differ dramatically because they contain transition metal atoms and varied surface terminations [3]. MXene and MAX phase are closely interconnected, as MXene is obtained from MAX phase materials by a specific etching procedure. MAX phases are compounds composed of layers with a particular formula of M_n+1_AX_n_ [[4], [5], [6], [7]]. In this particular context, the symbol “M" is used to indicate a transition metal. The symbol “A" is used to signify an element from either group 13 or group 14 of the periodic table, generally aluminum or silicon. The symbol “X" is used to denote either carbon or nitrogen. The value of “n" typically falls within the range of 1–3. The materials have a stratified composition similar to graphite, with alternating transition metal carbides or nitrides and A-element layers. MXenes have been identified as highly promising fire-retardant compounds, especially when included in polymer composites. Their efficacy can be due to several crucial characteristics: exceptional thermal stability, which slows down the decomposition of polymers; a barrier effect that creates a protective layer to impede the flow of heat and oxygen; and the facilitation of char formation, which serves as an insulating barrier during burning. In addition, MXenes can suppress free radicals in the gaseous state, further preventing the spread of flames. When these features are paired with their capacity to work together with other flame retardants, they greatly improve the ability of polymer composites to withstand fire [8].

MXenes are synthesized by selectively eliminating the A-element layers from MAX phase materials via an etching procedure [9,10]. A powerful etching agent, like hydrofluoric acid, carefully removes the A-element layers while leaving intact the transition metal carbide/nitride layers [1]. MXenes are formed as a layered structure of transition metal carbides or nitrides. MXenes possess several characteristics inherited from their parent MAX phases, including exceptional electrical conductivity, favorable mechanical properties, and remarkable stability at high temperatures [11]. Nevertheless, MXenes frequently demonstrate improved characteristics due to their flat structure and enlarged surface area, achieved by eliminating the A-element layers [12]. Because of their affinity for water, MXenes may easily be dispersed in water-based and polar organic solvents to create stable suspensions in colloids [13]. These solutions can then be filtered to produce films not attached to any substrate or spray-coated to make transparent conductive coatings [14,15]. This offers a broader range of potential applications for this group of materials. The early investigation of Ti_3_C_2_ [16], the first MXene, focused on its electrochemical characteristics in batteries and supercapacitors [17]. Since its discovery, MXene has attracted considerable attention in the scientific community because of its exceptional features and promising applications. Over the past few years, more than twenty-four MXenes have been identified [18], along with numerous more applications.

The construction of resilient and adaptable 3D MXene frameworks is still difficult because of feeble inter-sheet contacts. In response to this issue, a range of strategies have been devised. MXene/bio-based polymer aerogels utilize natural polymers such as cellulose to construct robust 3D structures that exhibit exceptional conductivity and mechanical resilience. Illustrative instances comprise cellulose/MXene aerogels and composites fabricated from discarded cotton fibers. Conversely, MXene/synthetic polymer aerogels utilize substances like polyimide to improve mechanical characteristics, inhibit oxidation, and reduce the clustering of nanosheets. Radial ice-templating and polyimide nanofiber composites enhance the pliability and compressibility of materials. Furthermore, including metal ions in MXene aerogels can greatly improve their structural stability and longevity. Gelation methods utilizing divalent ions such as Mg2+ and Zn2+ lead to the production of aerogels that exhibit enhanced durability and the ability to be easily scaled up. The sophisticated aerogels based on MXene have potential applications in various domains, including electromagnetic control, supercapacitors, sensors, solar steam generators, and batteries. These applications benefit from the combined features of the components [19].

MXenes exhibit distinctive characteristics, including exceptional mechanical durability, substantial surface area, and commendable chemical stability [20,21]. These features render them appropriate for diverse applications in many fields. MXenes exhibit exceptional electrical conductivity, making them auspicious materials for use in electronics and energy storage systems [22] and electromagnetic interference shielding [23]. They can be employed as conductive additions in composites or as active substances in electrodes for electromagnetic interference shielding, batteries and supercapacitors [24]. MXenes demonstrate a notable level of specific capacitance [25], making them very suitable for energy storage, including batteries and supercapacitors. Their expansive surface area enables effective ion adsorption and desorption, increasing charge storage capacity and accelerated charging/discharging rates [26]. MXenes have demonstrated favorable catalytic efficacy for many processes, such as hydrogen evolution reaction (HER) [27,28], oxygen reduction reaction (ORR) [29], and water splitting [30]. Due to their adjustable surface chemistry and many active sites, they are highly desirable catalysts for energy conversion applications and environmental cleanup [27]. MXenes have considerable potential in biomedical applications, such as drug administration, biosensing, and bioimaging, due to their biocompatibility, huge surface area, and ability to be functionalized with biomolecules [31]. These entities can be manipulated to selectively attack particular cells or tissues, rendering them highly favorable options for precise medication transportation systems [32]. MXenes have been studied for their potential in environmental remediation, specifically for their water purification applications and contaminants' elimination [33,34]. Due to their large surface area and ability to absorb chemicals, they are very effective at removing pollutants from water and other environmental materials [35]. MXenes can undergo thin film processing to provide exceptional transparency and flexibility [36]. MXenes are suitable for incorporating flexible and transparent electronic devices, including wearable technology, flexible screens, and transparent electrodes [37]. MXenes have a high level of electrical conductivity and may efficiently reduce the strength of electromagnetic radiation, making them suitable for electromagnetic interference (EMI) shielding [38]. They serve as constituents in polymer composites to enhance electromagnetic interference (EMI) shielding [39]. As a result, lightweight and efficient shielding materials are created for electrical gadgets, telecommunications equipment, and automotive parts.

This review paper aims to compile and integrate current research findings, methodology, and breakthroughs about MXenes. It offers a thorough summary of the present understanding of the topic, encompassing significant findings, obstacles, and upcoming developments. Additionally, it emphasizes discrepancies and constraints in existing knowledge, identifying areas that require further inquiry. Additionally, it functions as a guide for future research endeavors by proposing possible routes, tactics, and goals. This review paper serves as an instructive reference for researchers, students, and practitioners who have a keen interest in MXenes. This resource offers fundamental understanding, theoretical structures, and practical perspectives on the combination, characteristics, and uses of MXenes.

## 2 Fundamentals of MXenes

### 2.1 Structure of MXene

MXenes possess distinctive properties and have a wide range of uses due to their layered composition of transition metals and carbon or nitrogen atoms. Enhancing the abilities of MXenes by modifying and functionalizing them establishes them as a crucial material for advancing nanotechnology and materials research.

#### 2.1.1 Crystallographic structure

MXenes are created by removing the ‘A' ions from groups III-IV of the periodic table, specifically from MAX phases. These phases have a hexagonal P63/mmm structure and can be obtained by various methods. The leaching process leads to preserving the hexagonal structure in two-dimensional MXenes [40]. Alloy MXenes, including a combination of M′ and M″ ions, can display organized patterns within the layers or inside the plane [41]. If there is a substantial difference in the sizes of the ions, they may form a monoclinic structure. Exfoliation [42], typically achieved through HF treatment, disrupts the less strong M-A bonds [43], creating surface vacancies. These vacancies allow for the adsorption of OH, O and F groups, which stabilize the octahedral structure of MXenes.

**Fig. 1:**
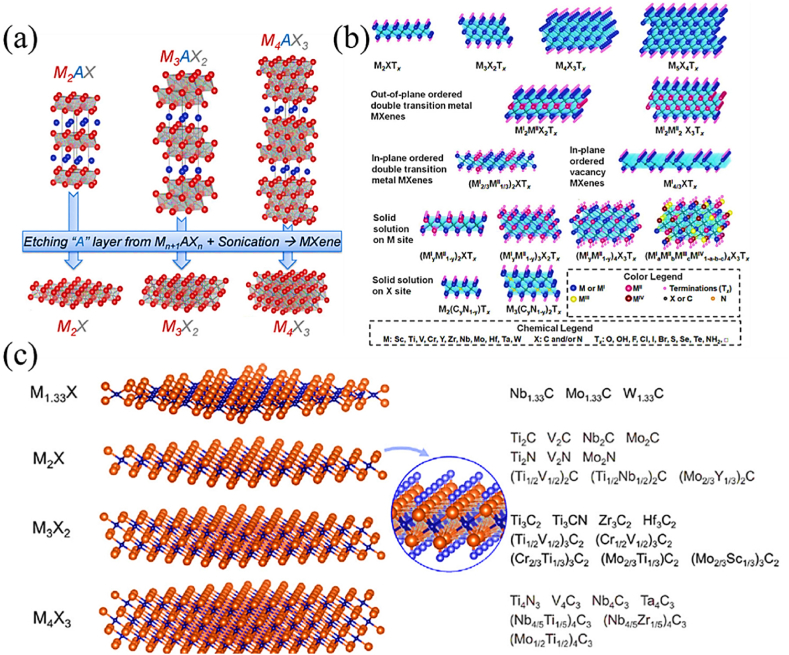
minations, X is either N or C, and M is an early transition metal. The range of the n value is 1–4. One, two, or more transition metal atoms can occupy the M sites to form ordered structures or solid solutions. (a) MAX phase structures and the MXenes that correspond with them. (b) Typical MXene structures and mixtures. (c) The structural and molecular formula of MXenes, were found experimentally.

Fig. 1 illustrates the synthesis method and the resulting layered structure of MXenes. The graphic depicts the sequential etching process, in which the elimination of the A element from the MAX phase results in the creation of layered MXenes. These MXenes possess several surface terminations that substantially impact their chemical and physical characteristics.

Source [44].

#### 2.1.2 Electric structure

Metalic MXenes without surface functionalization have Fermi energy on M ions' d-bands. In Sc_2_CT_2_ and M_2_CO_2_ (M = Zr, Hf, Ti), functionalization with O, F, or OH ions shifts the Fermi energy into the gap between X p and M d bands, creating semiconducting MXenes. Single-layer MXenes can be semiconducting, while double- and triple-layer ones are metallic. Semi-metallic behavior occurs when M ions reach the Γ point, and SOC creates a gap [45]. Topological semi-metallic or insulator compounds, such as M_2_CO_2_ (M = Mo, W) and M_2_′M″CO_2_ (M′ = W, Mo; M″ = Zr, Hf, Ti), display conducting edges and protection against nonmagnetic impurities [46].

#### 2.1.3 Magnetic structure

The magnetism of MXene is determined by band splitting, spin-orbit coupling, and electron states, all of which have significant roles. MXenes that include chromium (Cr) or manganese (Mn) frequently exhibit magnetic ordering [47]. Magnetism arises when nonbonding d orbitals, which are positioned between anti-bonding orbitals and bonding, cross the Fermi level. The anticipated magnetic MXenes consist of M_2_× (where M represents V, Ti, Ni, Mn, Cr, and X represents N, C) [48]. The crystal field influences the division of energy bands and spin bands, resulting in the emergence of metallic, half-metallic, or semiconducting characteristics. Most compounds have antiferromagnetic properties, whereas compounds containing Mn display ferromagnetic properties. The compound Hf_2_VC_2_F_2_ exhibits noncollinear ordering due to spin-orbit coupling [49].

**Fig. 2:**
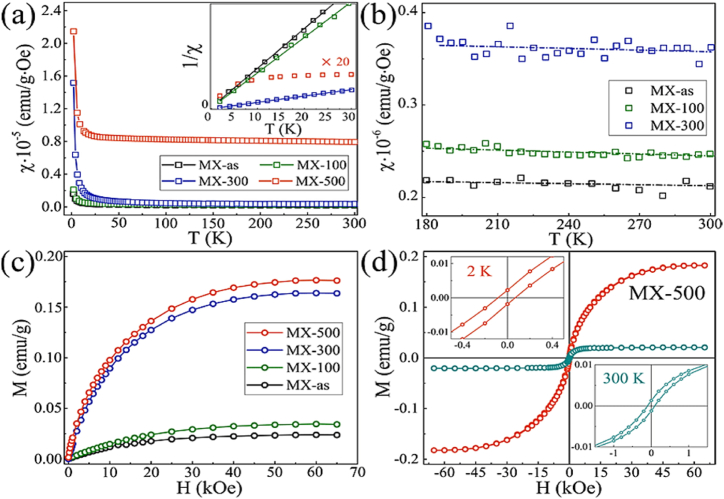
Magnetic characteristics of MXene.

Fig. 2 depicts the hierarchical arrangement of MXenes, which consist of transition metal layers alternating with nitrogen or carbon layers. The bonding within each layer is robust, but the cohesion between layers is maintained by weaker van der Waals forces. It emphasizes the magnetic characteristics of transition metal atoms (such as V, Cr, Ti), demonstrating several forms of magnetic arrangement: ferromagnetic (with parallel spins), antiferromagnetic (with alternating spins), and the impact of surface terminations like –O, –F or –OH on magnetic properties. The figure additionally presents a diagram of the electronic band structure, illustrating spin-polarized bands essential for spintronics. It also shows the impact of interlayer magnetic interactions on the overall magnetic behavior, highlighting the complex connection between the electronic, magnetic and structural properties of MXenes.

Source [50].

### 2.2 General characteristics of the MXenes

Table 1 presents a thorough summary of the physical properties of MXene materials, emphasizing the distinct features that set them apart from other two-dimensional materials. MXenes have attracted considerable attention since their discovery, thanks to the outstanding physical features that drive their numerous applications. MXenes provide desirable characteristics such as strong electrical conductivity, impressive mechanical strength, exceptional thermal stability, and adjustable surface chemistry, which make them well-suited for various technical and industrial applications.

**Table 1:** Physical property table of MXene [51,52].

Property	Value/Range	Description
Chemical Formula	M_n +1_X_n_ T_x_	M represents a transition metal (e.g., Nb, Ti, V), X is carbon and/or nitrogen, and T_x_T_x_T_x_ represents surface terminations (e.g., OH, F, O).
Crystal Structure	Hexagonal	MXenes have a layered structure similar to graphene, with transition metal layers alternating with carbon/nitrogen layers.
Thickness	1–3 nm (single layer)	MXenes are typically a few nanometers thick per single layer.
Lattice Parameter	∼3 Å (a-axis)	The lattice parameter varies slightly depending on the specific MXene composition.
Density	2.3–4.5 g/cm³	Depends on the specific composition and surface terminations.
Electrical Conductivity	1500–8500 S cm^−1^	MXenes exhibit high conductivity, which is attributed to their metallic properties.
Thermal Conductivity	10–80 W/m·K	Thermal conductivity varies widely depending on composition and orientation.
Young's Modulus	20–500 GPa	Indicates the stiffness of MXenes, which varies with composition and structure.
Mechanical Strength	0.3–1 GPa	Tensile strength varies significantly based on MXene type and synthesis method.
Surface Area	10–150 m^2^/g	High surface area, beneficial for applications like energy storage and catalysis.
Optical Properties	Tunable optical bandgap	MXenes display plasmonic characteristics and demonstrate adjustable optical properties based on their composition and surface terminations.
Magnetic Properties	Can be paramagnetic, ferromagnetic, or antiferromagnetic	The magnetic properties of MXenes strongly rely on the choice of transition metal and surface terminations.
Hydrophilicity	Highly hydrophilic	Due to surface terminations like OH groups, MXenes are generally highly hydrophilic, making them dispersible in water and other polar solvents.
Chemical Stability	Stable in inert atmospheres, but can oxidize in air	Stability is composition-dependent; for instance, Ti_3_C_2_ is known to be stable in inert atmospheres but can oxidize over time when exposed to air and moisture.

The table classifies various qualities, encompassing metrics such as thermal conductivity, mechanical strength, density, specific surface area, and electrical conductivity. Each attribute plays a crucial role in many applications: high electrical conductivity is required for energy storage devices and conductive films; strong mechanical strength promotes their utilization in structural materials; and high thermal conductivity makes them perfect for thermal management applications. This comprehensive exposition of the physical features of MXenes serves as a significant point of reference for researchers and engineers, enabling them to effectively choose and enhance MXene materials for applications. The research highlights the potential of MXenes to significantly transform fields such as electronics, energy storage, and thermal management by utilizing their distinct physical characteristics.

#### 2.2.1 Physical properties

Asymmetric O ion cages provide MXenes like Sc_2_CO_2_ ferroelectric polarisation. Transducer devices benefit from compounds with large piezoelectric coefficients (d11: 4–25 pVm^-1^) such as (3M 1/M′2/3)_2_CO_2_ (M′ = W, Mo; M″ = Sc, Y) [53]. Due to their Seebeck coefficient and high electrical conductivity, semiconducting MXenes like Sc_2_CT_2_ (T = OH, O, F) and M_2_CO_2_ (M = Zr, Ti, Hf) have strong thermoelectric power with ZT values around 1.1. Mo_2_C is superconducting with Tc < 4 K. Surface termination with H and Br can raise Tc to ∼13 K. The dielectric peaks below visible light of O-terminated Ti_3_C_2_ are affected by functionalization. Due to its high conductivity and wide absorption range (500–2700 nm), V_2_CT_x_ (T = OH,O, F) is ideal for conductive transport electrodes [54]**.**

#### 2.2.2 Chemical properties

MXenes may be surface functionalized to change their surface dipole moments and work function, allowing for Schottky-barrier-free interfaces and gas sensor applications. They are perfect for HER/OER reactions and water splitting because of their huge surface area, which increases catalytic activity. For HER, O-terminated W_2_CO_2_ and Ti_2_CO_2_ are especially useful. MXenes increase catalytic efficiency with metal ions, such as Fe, or hybrid structures, such as Ti_3_C_2_/g-C_3_N_4_ [55]**.** MXenes possess exceptional conductivity and a large surface area, making them highly attractive for energy storage. Functionalization can modify ion diffusion barriers, hence improving the efficiency of capacitors and batteries. Ti_2_CT_2_/graphene heterostructures, for example, provide greater diffusion barriers for Li ions [56]**.** Moreover, MXenes effectively aid in the diffusion of Na and K ions, and monolayer Ti_2_C is appropriate for hydrogen adsorption to produce hydrides.

#### 2.2.3 Electronic properties

MXenes possess exceptional two-dimensional electronic conductivity, rendering them very adaptable for a wide range of applications including electromagnetic interference shielding and energy storage. Moreover, their intrinsic characteristics render them very suitable for utilization in energy conversion apparatus such as solar cells. Surface chemical modification is essential for enhancing the electronic conductivity of MXene, which is vital for further enhancing its capabilities. Density functional theory simulations demonstrate a strong link between the surface terminations of MXene and the density of states at the Fermi level. Characterizing electronic qualities entails evaluating states' partial and total density, band structure, band gap, and electronic distribution [57]**.** The electrical properties of MXenes have been thoroughly investigated through theoretical and experimental research. These studies have shown that most MXenes have metallic or semimetallic behavior, while smaller MXenes have semiconductor capabilities. Three primary methodologies are typically employed to initially evaluate these electrical properties based on fundamental concepts. The fixed basis set method involves expanding the wave function using a predetermined set of essential functions, which can consist of linear combinations of Gaussian orbitals, atomic orbitals or plane waves [58]**.** The partial wave approach utilizes partial waves as the primary foundation for expanding the wave function. Commonly employed techniques in this context encompass the augmented plane wave method and the Rostoker- Korringa-Kohn approach. Determining partial waves in these procedures solely depends on the energy and potential [59]**.** The combined approach integrates aspects from both fixed basis set and partial wave methods, selecting components according to the individual demands of the case. It frequently employs the Rostoker-Korringa-Kohn approach while capitalizing on the benefits of fixed or partial wave basis sets. This technique relies on the assumption of spherical symmetry for the potential within nuclei and a consistent profile in the interstitial regions between nuclei. Every strategy has distinct benefits and is chosen depending on the circumstances being considered.

#### 2.2.4 Electrical properties

MXenes have attracted considerable attention since their discovery in 2011 because of their exceptional characteristics, including outstanding electrical conductivity, lightweight, flexibility, transparency, adjustable surface chemistry, and ease of solution processing. As a result, there has been significant investigation into the possible uses of these materials in many areas, such as shielding against electromagnetic interference, creating flexible optoelectronic devices, developing sensors, and producing thermal warmers. MXenes demonstrate higher electrical conductivity than other 2D materials, such as graphene, surpassing reduced graphene oxide (rGO) sheets by a factor greater than 10 [60]. Among MXenes, Ti_3_C_2_ is particularly notable for its exceptional application performance, including batteries, water purification, supercapacitors, sensors and flexible electronics composites. The inclusion of galleries in electrically conductive MXenes enables the incorporation of various ionic and molecular species, thanks to the surface terminations with OH, O, and F groups, resulting in the hydrophilic nature of MXenes [61]**.** Recent progress has shown that MXene films have outstanding performance in tensile strength, electrical conductivity, and electromagnetic interference (EMI) shielding properties. The electrical conductivity of MXenes can be affected by different external influences, including the conditions of fabrication, the source material of MAX, treatments after etching, and ultrasonication. Prudent control of these parameters is essential for applications requiring substantial electrical conductivity. Research has demonstrated that MXenes produced from different carbon precursors display varied levels of electrical conductivity and colloidal stability. For example, MXenes produced from carbon precursors based on graphite exhibit superior electrical conductivity and greater stability when compared to MXenes formed from precursors based on TiC or carbon lampblack. The advantages of creating electrically conductive materials are clear, as Ti_3_C_2_Tx MXene films surpass graphene in terms of electrical conductivity and EMI shielding effectiveness. MXenes possess exceptional electrical conductivity, transparency, and dispersion characteristics, rendering them well-suited for integration into polymer matrices for many applications. Gaining insight into the correlation between the structure and properties of MXenes is crucial for maximizing their electrical characteristics. The metallic characteristics of MXenes have been explained using theoretical models and experimental evidence. Effective management of the synthesis process is essential to minimise errors that may impact the electrical characteristics of materials based on MXene [62]**.** To summarise, MXenes demonstrate remarkable electrical characteristics, which make them highly attractive for various applications. Their distinct amalgamation of characteristics and continuing research endeavors continue to broaden their potential in several domains, ranging from energy storage to biosensing and beyond.

#### 2.2.5 Optical properties

The outstanding optical, electrical, elastic, mechanical, and transport capabilities of two-dimensional MXenes have garnered substantial attention in various disciplines, including optoelectronics, supercapacitors, solar cells, and biomedicine. Due to their distinctive attributes, they are extremely well-suited for a diverse array of uses. MXenes demonstrate exceptional non-linear optical characteristics, rendering them highly favorable for applications in ultrafast photonics. The optical characteristics of MXenes are intricately connected to their electrical and structural features. To evaluate their optical properties, it is essential to comprehend the dielectric constant of MXenes, represented as *ε*(ω). The constant consists of two components: a real part, εr(ω), and an imaginary part, εi(ω). Both components are crucial for understanding the optical properties of the material. The dielectric constant is calculated based on the electric susceptibility, χ(ω), and the optical conductivity is determined by the product of the angular frequency ω, the vacuum permittivity *ε*0, and the electric susceptibility χ(ω). In addition, the refractive index of the material, denoted as n + ik, is linked to the complex dielectric constant, where k represents the extinction coefficient associated with the absorption properties [63]**.** The presence of functional groups greatly influences the electrical and band structures of 2D MXenes, hence impacting their optical features. The presence of intraband and interband electronic transitions considerably affects the imaginary component of the dielectric constant in materials that contain transition metals. The collective interbond electronic transitions mostly determine the absorption spectrum and absorption coefficient of MXenes. MXenes with hydroxylated or fluorinated terminations tend to gain a single electron, which leads to similar optical characteristics in the visible range of the electromagnetic spectrum. Conversely, MXenes with oxygen terminations tend to gain two electrons, resulting in distinct variations in their optical characteristics upon exposure to visible light. For example, Ti3C2 with oxygen surface functionalization exhibits enhanced absorption in the visible spectrum, while fluorinated samples have inferior absorption capabilities [64]**.** Utilizing density functional theory (DFT), computational studies enable the estimation and analysis of the influence of oxygen atoms on the partial density of states (PDOS) and total density of states (TDOS). This provides insights into the fundamental mechanisms that determine the optical characteristics of MXene [65]**.** MXenes have captivating optical characteristics affected by surface functionalization and electronic structure. These qualities have great potential for many applications in photonics and other field [66,67].

#### 2.2.6 Magnetic characteristic

The magnetic properties of MXenes are greatly affected by surface stretching and covalent bonding between the M and X elements. This generally leads to a lack of magnetic properties due to the strong bonds and high electron density around the Fermi Zones (FZ). However, specific MXenes display magnetic properties due to surface stretching inside the plane or changes in electron density caused by d orbitals and other influences. Cr_2_C, Cr_2_N, Ta_3_C_2_, and Cr_3_C_2_ exhibit ferromagnetic (FM) behavior, whilst Ti_3_C_2_ and Ti_3_N_2_ display antiferromagnetic (AFM) qualities [[68], [69], [70]]**.** Ti_2_C and Ti_2_N, on the other hand, demonstrate half-metallic FM characteristics. The characteristics can be modified by surface functionalization using elements such as OH, Cl, F, and O, which can occasionally transform AFM states into FM states, as shown in Mn_2_C monolayers [71]**.** In addition, the application of tensile strain can cause transitions between ferromagnetic (FM) and antiferromagnetic (AFM) states or improve the ferromagnetic properties [72]. Functionalized MXenes show promise for flexible electronics and spintronics applications due to their ability to be modulated by small electric fields, which can also affect magnetism. Functionalized MXenes, such as Hf_2_VC_2_F_2_, demonstrate multiferroic characteristics by coupling ferroelectricity and magnetism [73]**.** These materials may retain their magnetic properties at elevated temperatures due to substantial energy gaps between ferromagnetic (FM) and antiferromagnetic (AFM) states [74]**.** Research on chromium-based MXenes has demonstrated a wide range of magnetic properties depending on the surface groups present. In contrast, titanium-based MXenes such as Ti_2_C and Ti_2_N maintain their half-metallic ferromagnetic features even when subjected to stress. MXenes possess adjustable magnetic characteristics, making them well-suited for electronics, spintronics, and photocatalysis. Additionally, their auxetic, piezoelectric, and ferroelastic capabilities further enhance their usefulness in advanced technological domains [75]**.**

#### 2.2.7 Mechanical characteristics

MXenes, a type of two-dimensional (2D) materials, are receiving considerable interest because of their strong M − N (metal-nitrogen) and M − C (metal-carbon) bonds, which contribute to their mechanical properties. MXenes have mechanical qualities that surpass those of numerous other materials due to the presence of these bonds. Simulation studies suggest that MXenes exhibit elastic properties that are approximately twice as high as those of MAX phases and comparable to other 2D materials, such as CdS_2_. However, the elasticity of MXenes is still two to three times lower than that of graphene [76]**.** However, MXenes demonstrate remarkable flexibility, reaching a maximum value of 1050 GPa, which makes them very suitable for application as reinforcing elements in composites [77]**.** Their interaction with polymer matrices surpasses that of graphene, mostly due to the presence of functional groups, which greatly enhance their effectiveness in composites.MXene thin films, namely those composed of titanium, exhibit hydrophilicity, as evidenced by contact angles ranging from 25 to 40° [78]**.** Studies suggest that raising the number of layers reduces Young's modulus for MXene nitrides and carbides. However, nitride-based MXenes exhibit greater values. The occlusion of endpoints considerably impacts the C11 constants and leads to higher critical deformation levels, making MXenes potentially advantageous for foldable electronics. However, assessing their mechanical properties can be hard. The practical Young's modulus of a Ti_3_C_2_T_x_ monolayer was determined using nano-indentation, a commonly used technique for measuring the mechanical properties of 2D materials. The obtained value was 333 ± 20 GPa [79]**.** The value for Ti_3_C_2_O_2_ is around 386 GPa, more than that for graphene oxide and MoS2. Nevertheless, the accurate forecasting of MXenes by mechanical means is impeded by challenges in monitoring surface conditions, dimensional vacancies, and interactions between composite materials. Therefore, additional study is required to enhance production procedures and better understand the nature of defects [80]**.**

#### 2.2.8 Thermal characteristics

Thermal stability and conductivity are essential factors in evaluating the performance of MXenes. Research has demonstrated that the ability of MXenes such as Ti_3_C_2_T_x_, Nb_2_CT_x_, and Mo_2_CT_x_ to withstand high temperatures up to 1500 °C is greatly influenced by their composition and the method used to remove certain layers [81,82]**.** Ti_3_C_2_T_x_ showed higher thermal stability than Nb_2_CT_x_ and Mo_2_CT_x_, further improved stability by employing a reduced range of HF during the etching process. The V_2_CT_x_ MXene exhibited minimal oxidation when exposed to oxygen above 300 °C, while its microstructure remained intact in nitrogen or hydrogen gas [83]**.** However, the structure of the MXene degraded at elevated temperatures in the presence of air. A study on the thermal conductivity (TC) of Ti_3_C_2_T_x_ films was conducted, and it was discovered that both the inherent TC and the thermal resistance between flakes contribute to the overall TC. The increased lateral dimensions of Ti_3_C_2_T_x_ flakes (D) and the decreased contact resistance between flakes enhance thermal conductivity (TC). Phonon scattering and interface thermal resistance are crucial elements that significantly impact thermal conductivity [84]**.** A following study discovered that M_2_PC ceramics (where M represents V, Nb, or Ta) exhibit high Debye temperatures (θD), suggesting the presence of rigid lattices and hence a favorable TC (thermal conductivity) [85]**.** The mentioned study on the thermal conductivity (TC) of Sc_2_CT_2_ (T = OH, F) and O-functionalized M_2_CO_2_ (M = Zr, Ti, Hf) also found that Sc_2_CF_2_ exhibited a higher TC compared to conventional conductors such as silver. Hf_2_CO_2_ exhibited superior thermal conductivity (TC) compared to MoS_2_ and phosphorene, with significant thermal expansion coefficients and carrier mobility, rendering it well-suited for electrical devices. Ti_2_CO_2_ exhibited a lower thermal conductivity (TC) but better carrier mobility than Hf_2_CO_2_. Mo_2_C demonstrated a thermal conductivity (TC) of around 48 Wm^−1^K^−1^, which rose as the temperature increased. It also possessed a specific heat of 290 JKg^−1^K and a thermal expansion coefficient of 2 × 10^−6^ K^−1^ at room temperature [86]. The findings emphasize the favorable thermal characteristics of MXenes for diverse technological applications.

#### 2.2.9 SEM analysis

**Fig. 3:**
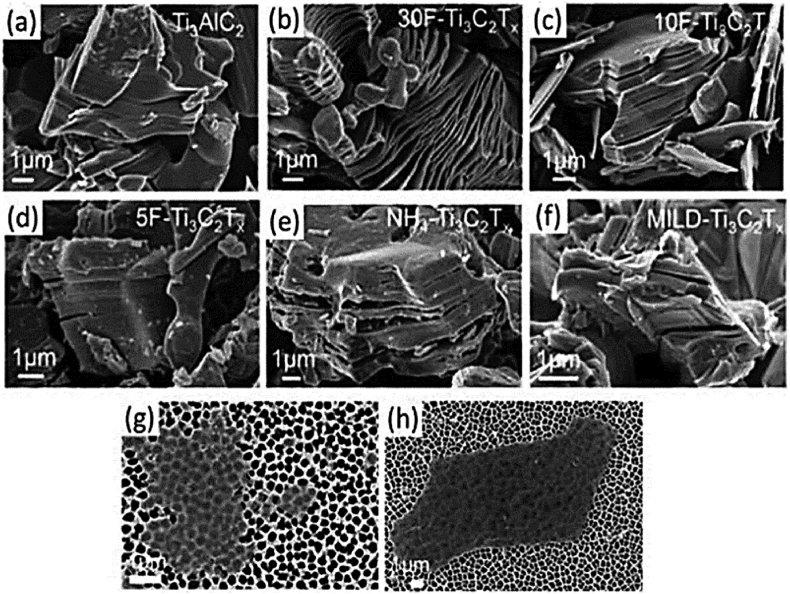
The provided images are scanning electron microscope (SEM) images of MXenes and MAX powders that have been produced using etching processes under various conditions. Scanning electron microscope (SEM) images of (a) Ti_3_AlC_2_ (MAX) powder displaying a dense layered arrangement and (b) multilayered Ti_3_C_2_T_x_ powder produced using hydrofluoric acid (HF) synthesis at 30 %, 10 %, and 5 % weight percentages. An accordion-like morphology was only detected after etching in a solution containing 30 wt percent HF or higher concentrations of HF. The NH_4_– Ti_3_C_2_T_x_ powder, synthesized using ammonium hydrogen fluoride and the MILD method (etched with LiF in HCl), exhibits minimal opening of MXene lamellas, comparable to what is reported in 5F Ti_3_C_2_T_x_. The SEM photos show individual MXene flakes on a porous alumina substrate that were etched using a combination of 5 % HF and MILD methods [87].

Occasionally, the oxidation level is too minimal or inadequate to be detectable using X-ray diffraction (XRD). Scanning electron microscopy (SEM) is employed to observe the structure of MXenes. However, the appearance of etched samples may differ. As the concentration of HF declines, the “accordion” structure gradually disappears, and the multilayer MXene becomes more similar to the normal MAX structure seen in Fig. 3. The presence of oxide nanoparticles, which are indicative of surface oxidation and a solid etching process, can also be observed on the exterior of the layers, as demonstrated by scanning electron microscopy (SEM). Fig. 3 displays scanning electron microscope (SEM) images of the compact layered Ti_3_AlC_2_ (MAX) powder and the MAX phase. The images show the multilayered Ti_3_C_2_T_x_ powder created with different weight percentages of hydrofluoric acid (HF): (b) 30 %, (c) 10 %, and (d) 5 %. The distinctive accordion-like morphology becomes apparent only after etching with HF solutions with 30 wt percent or higher concentrations. The image (f) displays MXene that has been etched using the MILD method with LiF in HCl. Both photos demonstrate a restricted opening of the MXene layers, like what is observed with Ti₃C₂Tₓ etched using 5 wt% HF. Image (e) displays a powder composed of many layers of NH₄–Ti₃C₂Tₓ, created using the synthesis process involving ammonium hydrogen fluoride. Moreover, SEM pictures (g) and (h) depict individual MXene flakes that were etched using the MILD technique and five-weight percent HF on a porous alumina substrate, respectively.

### 2.3 Synthesis method

**Fig. 4:**
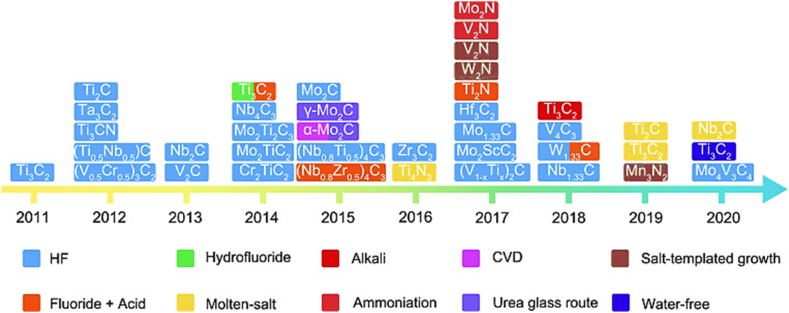
MXenes synthesis routes timeline in the last decade.

Reproduced with permission [88].

Fig. 4 shows the MXenes synthesis routes timeline in the last decade. In order to create 2D flakes with the general formula M_n+1_X_n_T_x_ (n = 1, 2, 3), MAX phases are converted into MXenes by eliminating the “A" ingredient. In this context, M represents metallic elements such as Titanium (Ti), Niobium (Nb), Chromium (Cr), Molybdenum (Mo), and others. X refers to carbon and/or nitrogen, while Tx indicates surface terminations such as Fluorine (F), Hydroxyl (OH), or Oxygen (O). To selectively remove “A", one can employ either top-down procedures such as liquid-based etching or bottom-up methods such as CVD or carbonization. Several synthesis techniques encompass chemical vapor deposition (CVD), wet selective etching, template approaches, plasma-enhanced pulsed laser deposition (PEPLD) and intercalation.

#### 2.3.1 Wet chemical etching

**Fig. 5:**
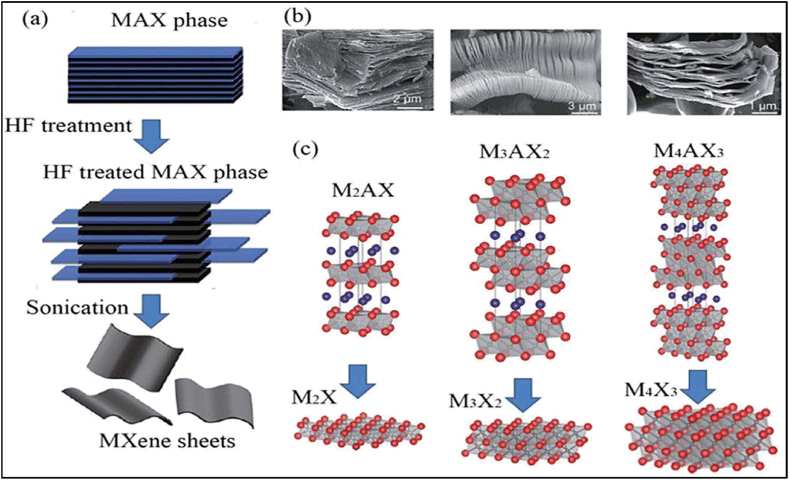
(a) Three different MXene (non-terminated) structures—M_2_X, M_3_X_2_, and M_4_X_3_—were first described. (b) Ti_2_AlC, Ti_3_AlC_2_, and Ti_4_AlC_3_ SEM images following HF treatment (from right to left). (c) Schematic representation of the synthesis of MXenes from MAX phases [87].

2D MXenes are synthesized using the process of wet selective etching, in which the “A" layer is selectively removed from MAX phases (Fig. 5) at room temperature using an etchant such as aqueous HF [86]**.** Hydrofluoric acid (HF) disrupts the less strong metal-to-metalloid connections, making it easier to separate the layers using ultrasonication, centrifugation, or filtration techniques. The resulting MXenes, which have a similar appearance to exfoliated graphite, are subsequently rinsed with deionized water until they reach a final pH range of 4–6 [89]**.** By manipulating the concentration of HF, etching time, and ultrasonic time, this technique can generate MXenes with diverse surface terminations such as OH, O, and F and different morphologies. These method changes allow for achieving over 20 distinct MXene compositions and stackings.

Ultrasonication greatly decreases the time required for etching Ti₃AlC₂ powders with 49 % HF from 24 to 4 h by fragmenting MXene flakes into smaller sheets and aiding in etching [90]**.** A following study discovered that the process of ultrasonication-induced exfoliation of Ti₃Si₀.₇₅Al₀.₂₅C₂ is influenced by the type of solvent used and is ineffective for pure Ti₃SiC₂. The exfoliated sheets have a thickness of around 4 nm and lateral diameters ranging from 100 to 200 nm [91]**.** Intercalation causes an increase in the lattice parameter c, which is influenced by the size of the solvent's molecules. The range of Ti₃C₂T_x_ is from 0.7 Å (sodium sulfate) to 15.4 Å (DMSO) [92,93]**.** The presence of ambient moisture progressively enhances this parameter as time elapses. Titanium carbide (Ti₃C₂T_x_) can be synthesized at room temperature using either HF or in-situ HF with various intercalants [94]**.** Wet chemical etching produces MXenes that exhibit elevated electrical conductivity and reduced flaws but at the expense of employing the hazardous and cancer-causing substance hydrofluoric acid (HF). Various fluoride salts (LiF, NaF, KF, NH₄F) have been investigated as alternative etching techniques utilizing HCl [95,96]**.**

#### 2.3.2 Urea glass route

Nanocrystalline material with a single-phase composition Mo_₂_N and Mo_₂_C MXenes were produced from the synthesis of MoCl₅ using a urea glass method, with different ratios of urea to the metal precursor. Mo-orthoesters are formed by dissolving MoCl₅ in ethanol and reacting it with urea. These orthoesters then undergo gelation and calcination at a temperature of 800 °C in an environment devoid of reactive substances, forming a silver-black precipitate [97]**.** Urea functions as a dual-purpose compound, serving as both a carbon and nitrogen source and a stabilizing agent. This environmentally friendly process generates MXenes with substantial surface areas, making them highly efficient as catalysts [98]**.**

#### 2.3.3 Chemical vapor deposition

Chemical vapor deposition (CVD) is a highly successful method for manufacturing 2D materials of excellent quality that possess extensive surface areas. The following study synthesized 2D ultrathin α-Mo₂C crystals by utilizing methane as the source of carbon [99]**.** They achieved this by placing a layer of Cu foil on top of Mo and heating it to a temperature of 1085 °C. This process resulted in forming a Mo-Cu alloy and producing MO₂C crystals [100]**.** Regulating the methane concentration is necessary to avoid the creation of graphene. These crystals, which are only a few nanometers thick, have a size of over 100 μm and are highly stable [101]**.** CVD also generates other carbide crystals, such as tungsten and tantalum carbides. Wang et al. employed a diffusion-coupled Ta-Cu bilayer with acetylene, ammonia, and boron powder as precursors to synthesize TaC, TaN, and TaB. This process improved the characteristics of the Cu-matrix, making it well-suited for applications in sensors, batteries, hydrogen storage and catalysis owing to its excellent electrical conductivity.

#### 2.3.4 Molten salt etching

**Fig. 6:**
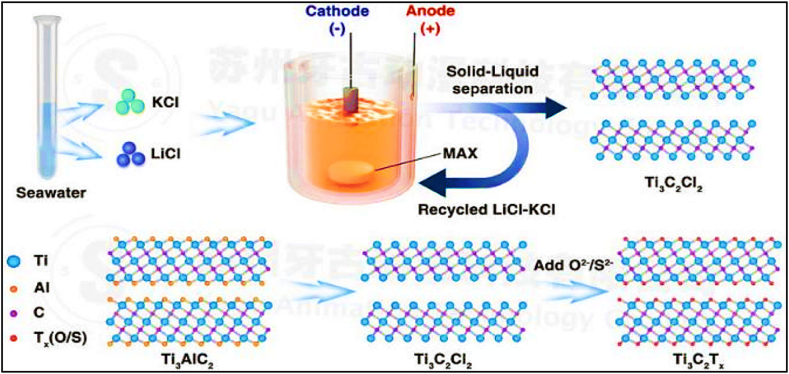
MXene synthesis by MS-E-etching from the MAX phase, then controlled surface terminations made possible by salt lakes' plentiful supplies of potassium and lithium. Notably, the process is more controllable due to using eutectic salts, including KCl and LiCl, with a low melting point [134].

Etching the element “Al” from MAX phases is more straightforward than etching the element “N" because Ti_n+1_N_n_ has lower stability and cohesive energy than Ti_n+1_C_n_. The bonding between aluminum atoms is stronger in Ti_n+1_AlN_n_, necessitating more energy for extraction [102]**.** Nitride MXenes exhibit a greater density of states (DOS) at the Fermi level, which results in an increased metallic nature and enhanced electronic conductivity. These properties make them suitable for electrochemical capacitors, plasmonic materials, and metamaterial devices.Ti₄N₃T_x_ MXenes [103]**,** which are challenging to fabricate using wet etching, can be produced via a molten salt. Aluminum is removed from titanium aluminum nitride (Ti₄AlN₃) by employing a 1:1 mass ratio of fluoride salts (KF, LiF, NaF) at a temperature of 550 °C in an environment devoid of reactive substances [104]**.** Using tetrabutylammonium hydroxide (TBAOH), the MXene can be separated into a small number or even just one layer of flakes [105]. Fig. 6 illustrates the process of submerging the precursor material into a molten salt bath. Halides such as fluoride or chloride salts are frequently employed and are subjected to high temperatures to form a reactive media. The molten salt acts as a substance that dissolves or corrodes, making it easier to selectively remove A layers from the MAX phase. The molten salt engages with the MAX phase within the schematic, commencing the etching procedure. The diagram depicts the process by which the molten salt specifically eliminates the layers containing the A element, thereby separating the MXene layers and causing the structure to exfoliate. This is demonstrated by the fragmentation of the MAX phase and the liberation of A elements into the liquid salt solution. Chloride salts-based etching is a technique employed to specifically eliminate components from a substance, especially during the manipulation of MAX phases. The utilization of chloride salts, such as sodium chloride (NaCl) or potassium chloride (KCl), enables effective etching by virtue of their capacity to generate volatile by-products with the A element in MAX phases, which may be readily eliminated from the material. The energy dynamics of this process are vital in selecting the suitable salt, as the decision relies on the particular A element found in the MAX phase. For example, materials that are more reactive or have lower melting temperatures may necessitate the use of salts with lower activation energies in order to achieve effective etching without the need for significant energy input. By judiciously choosing the chloride salt according to the properties of the A element, the etching process can be adjusted to achieve both high efficiency and precise results.

#### 2.3.5 Hydrothermal synthesis

The use of hydrofluoric acid in the etching process of MAX phases during MXene production may negatively impact the performance of lithium-ion batteries and supercapacitors [106]**.** Theoretically, alkali etching of Ti₃AlC₂ is feasible because of the amphoteric nature of Al. However, producing an oxide/hydroxide layer on Ti₃AlC₂ hinders kinetics [107]**.** The Bayer process resolves the issue by increasing alkali concentration and temperature. This leads to the dissolution of aluminum oxides/hydroxides and enhances the etching process [108]**.** Based on this inspiration, a hydrothermal method employs a solution of 27.5 M NaOH at a temperature of 270 °C to generate MXenes without fluorine, resulting in a 92 % weight yield of Ti₃C₂T_x_ [109]**.** The controlled reaction environment in hydrothermal synthesis makes it possible for almost all the reactants to be turned into the desired output. This is what makes the method famous for its high yield. This method is also great for making materials with certain electrical qualities, like high conductivity, especially in conductive oxides. Other ways of etching, like chemical and laser etching, have different benefits. Chemical etching can give moderate to high outputs depending on the materials and chemicals used. However, because the reactions are not as selective, they can also make unwanted by-products. Plasma etching, on the other hand, is very selective and gives you exact control. It often results in high yield with few residues, but it might need more work afterward. It is possible to make materials with high conductivity using hydrothermal synthesis. On the other hand, chemical etching can produce surfaces with different conductivity levels based on how well and evenly the process is carried out. Hydrothermal methods can change the electrical qualities of a material more easily than plasma etching, which tends to keep them the same. Overall, hydrothermal synthesis is a better method when high output and good conductivity are important. Other etching methods, on the other hand, may be better for certain tasks because they are more precise and selective.

#### 2.3.6 Electrochemical synthesis at room temperature

Electrochemical synthesis is a highly effective technique for generating MXenes without fluoride. Mono and bilayer Ti₃C₂T_x_ (where T represents O, OH, etc.) are produced through anodic corrosion of Ti₃AlC₂ in a binary aqueous electrolyte, resulting in a yield of over 90 % [110]**.** The procedure entails utilizing two Ti₃AlC₂ components as the anode and cathode within an electrolyte solution consisting of 1 M NH₄Cl and 0.2 M TMA·OH (pH > 9). Chloride ions cause rapid erosion of aluminum, whereas NH₄OH infiltrates and exposes the edges and surfaces of the anode. At room temperature, the anode delaminates after approximately 5 h when subjected to a low voltage of +5V [111]**.** This process results in the formation of Ti₃C₂T_x_ sheets, which are then filtered and ground. The result, devoid of fluoride, has exceptional areal capacitance (220 mF cm⁻^2^) for supercapacitors. This approach also effectively synthesizes additional MXenes such as V₂CT_x_ and Cr₂CT_x_. MXenes are adaptable materials that may adjust their surface properties, affecting their electrical and electrochemical characteristics [112]**.** This makes them highly promising for various applications, including batteries, capacitors, and other uses. Nevertheless, enduring issues such as restacking, limited flexibility, and inadequate stability remain. The characteristics of materials can be enhanced through doping and substitution techniques, resulting in the formation of hetero-MXenes. Various uses encompass energy storage, electrocatalysis, and sensors.

The potential chemical processes that occur during electrochemical etching include:

Ti_3_ AlC_2_ + 3e−+ 3Cl− ⇒ Ti_3_ C_2_ + AlCl_3_

Ti_3_ C_2_ + 2OH− − 2e− ⇒ Ti_3_ C_2_ (OH)_2_

Ti_3_ C_2_ + 2H_2_ O ⇒ Ti_3_ C_2_ (OH)_2_ + H_2_

#### 2.3.7 Alkali-based etching

A limited amount of research has investigated the process of alkali etching of MXenes. Alkali etching is possible because aluminum (Al) in the MAX phase forms strong bonds with alkali [113]. However, this process is limited by the formation of surface hydroxides. Li et al. conducted a study on the etching of Ti₃AlC₂ using NaOH [114], resulting in the production of Ti₃C₂T_x_ MXene with high purity and no fluorine. The etching process led to the termination of the MXene with -OH and = O groups [115]. In addition, they employed potassium hydroxide (KOH) and water to etch aluminum (Al) from titanium aluminum carbide (Ti₃AlC₂), resulting in the formation of titanium carbide hydroxide (Ti₃C₂(OH)₂), which can be readily separated into layers and reassembled [116]. This MXene possesses excellent electrical conductivity and hydrophilic properties, making it highly appropriate for various applications [117].

#### 2.3.8 Aqueous acid etching (AAE) method

MXenes are produced through the targeted removal of atomic layers from MAX phases. Mechanical exfoliation is used for some compositions, although it is not universally applicable because of the presence of strong metallic or covalent bonds [118]. One commonly used technique involves using hydrofluoric acid (HF) to etch the surface [112]; this technique incorporates functional groups that influence the characteristics of MXene. Tetrapropylammonium hydroxide (TPAOH) is employed throughout the intercalation process to assist in the separation of the layers. These layers are then isolated as MXene nanosheets using centrifugation. By performing high-temperature etching in an atmosphere rich in argon, MXenes like Ti_4_N_3_ can be produced from Ti_4_AlN_3_ [119]. It is essential to carefully regulate the temperature to prevent the creation of bulk compounds. Gaseous etchants have been substituted because they lack the capacity to remove atomic layers selectively, and inadequate temperature regulation can create the first MAX phase [120,121].

#### 2.3.9 In-situ HF-forming etching method

Using hydrofluoric acid (HF) to produce MXenes entails notable safety hazards. Hence, it is imperative to investigate alternate techniques to minimise the necessity of using direct HF solutions in the preparation procedure [122]. An encouraging strategy involves advancing in-situ HF-forming etching techniques, exhibiting exceptional etching outcomes. These procedures can be classified into two main groups: etching with fluoride salts/acids and etching with bifluoride salts. The etching of Al-containing MAX demonstrates that the effective attachment of F ions to the Al layer is crucial for achieving excellent etching results [123].

#### 2.3.10 Bifluoride salts etching method

A viable solution to mitigate the safety issues related to HF etching procedures is the utilization of the bifluoride salts etching technique. In 2014, Halim et al. utilised NH_4_HF_2_ etchant for the initial time to etch Ti_3_AlC_2_ MAX and eliminate Al layers. The successful isolation of individual Al atom layers was verified by STEM imaging, which showed a consistent arrangement of Ti and C atom layers with a significant distance between each layer. Feng et al. conducted further research comparing the etching effects of NH_4_HF_2_-etched Ti_3_C_2_ MXene with HF-etched Ti_3_C_2_. The XRD analysis revealed that Ti_3_C_2_ treated with NH4HF2 had a decreased angle for the (002) peak and an increased c lattice parameter compared to Ti_3_C_2_ treated with HF. The use of ammonium species (NH_3_ and NH_4+_) in the etching process enables the removal of MAX, while simultaneously dissolving the aluminium layer through intercalation [124].

The etching procedure using bifluoride salts involves the reaction of a compound called MAX with certain salts, such as NH_4_HF_2_, NaHF_2_, or KHF_2_. An investigation was conducted to optimise the etching process by studying the reaction conditions, specifically the reaction temperature, reaction time, and MAX size. A refined reaction condition was determined, utilizing a maximum particle size of 325 mesh, operating at 60 °C for 8 h. The MXene obtained demonstrated superior thermal stability compared to the MXene obtained using HF etching. NaHF_2_ and KHF_2_ were also investigated as etchants, necessitating a reaction time of over 8 h for the exfoliation of Ti_3_AlC_2_. The reaction process entails the displacement of distinctive peaks in XRD patterns and the creation of a structure like an accordion, which signifies the achievement of effective etching [125]. The chemical mechanism of the bifluoride salts etching approach can be described by equations that illustrate the transformation of Ti_3_AlC_2_ MAX into Ti_3_C_2_ MXene, resulting in the production of AlF_3_ and H_2_ gas. Despite the improved safety and greater quality of MXene products achieved by using bifluoride etchants instead of HF etching, additional investigation is necessary before these etchants may be widely implemented. Ultimately, the utilization of bifluoride salts etching technique offers a more secure substitute to HF etching for producing MXenes. Although this approach has significant benefits, further research is necessary to comprehensively comprehend and enhance its effectiveness. MXene compositions and application.

### 2.4 Challenges in MXenes synthesis

The synthesis techniques of MXenes are directly connected to their quality, beginning with the MAX phase precursor, which must be of high purity and devoid of any unreacted substrates and contaminants from mechanical milling. The process of etching the MAX phase material entails the removal of aluminum layers. Initially, this was done using poisonous hydrofluoric acid (HF). However, safer alternatives like the MILD approach and ionic liquids have been developed. It is worth noting that these alternatives have some limits in terms of scalability due to their cost. The grain size of the MAX phase is crucial. Larger grains lead to unetched MXenes that necessitate extra etching, whereas smaller grains are easily etched but may introduce contaminants and generate smaller flakes. The delamination process is influenced by the size of the grains, where larger grains require longer delamination periods while smaller grains produce larger flakes after sonication [126]. When characterizing MXenes, it is important to carefully analyze factors such as surface zeta potential, conductivity, and Raman and XRD examinations. This is because differing functionalities might result in similar spectra and patterns, making it difficult to follow changes in the chemical composition and dimensions of the flakes. The efficient elimination of residual substances produced during aluminum etching is crucial and can be accomplished when the pH of the solution reaches 6 or 7. However, significant concentrations of dispersed substances might make this process difficult, requiring additional procedures such as washing, centrifugation, or filtration [127]. The synthesis and stoichiometry of MXenes exhibit variations. For instance, Ti_3_C_2_T_x_ can be readily etched and delaminated using LiF/HCl, while Nb2CTx necessitates HF etching followed by TBAOH delamination. MXenes such as Cr_2_CT_x_, V_2_CT_x_, and Ti_2_NT_x_, which are challenging to etch, are typically composed of many layers because of their strong inclination to oxidize and limited stability. The problem of oxidation stability persists, as MXenes structures become unstable upon delamination and quickly oxidize under normal settings. Therefore, they must be stored at low temperatures in dark, oxygen-free environments [128]. Characterization techniques are influenced by rapid oxidation; materials must be analyzed immediately after manufacture to prevent alterations during preparation. The XRD and Raman patterns may exhibit variations due to oxidation, which can be alleviated by employing suitable laser intensities during Raman analysis. Surface chemistry alterations can be assessed using zeta-potential or XPS methods. Prompt analysis indicates significantly negative zeta-potential values and metallic bonding without metal oxides, while oxidized samples exhibit nearly zero zeta-potential and confirm the presence of metal oxides**.**

## 3 MXene-reinforced polymer composites

Numerous publications obtained from the Web of Science demonstrate the explosion of research on MXene-reinforced polymer composites over the last six years. This trend is demonstrated by the increasing interest in MXene-reinforced polymer composites and their wide range of possible applications. These composites have amazing qualities, including mechanical strength, thermal stability, and electrical conductivity, because of the special qualities of MXenes. These qualities can be precisely tuned by changing the composition, kind of polymer, and type of MXene.

### 3.1 Fabrication methods and processing

#### 3.1.1 Direct physical mixing

The predominant approach for fabricating MXene-reinforced polymer composites involves directly blending MXene with polymers. Melt blending is a technique used to combine MXenes with linear thermoplastic polymers such as polylactic acid (PLA), polypropylene (PP) and thermoplastic polyurethane (TPU). The polymer is heated until it reaches a molten condition and then thoroughly combined with MXene by continuously stirring. For example, MXene and TPU particles can be combined in an internal mixer at a temperature of 160 °C and a rotational speed of 40 rpm for a duration of 5 min prior to being compressed into samples [129]. Furthermore, the integration of MXene nanosheets and short aramid fibers (SAF) can be achieved through melt blending, resulting in the formation of reinforced composites known as Ti_3_C_2_T_x_/SAF/PP. Another technique is solution casting, which entails mixing the polymer matrix and filler in a solvent, then casting the mixture into a mold and allowing the solvent to evaporate.

**Fig. 7:**
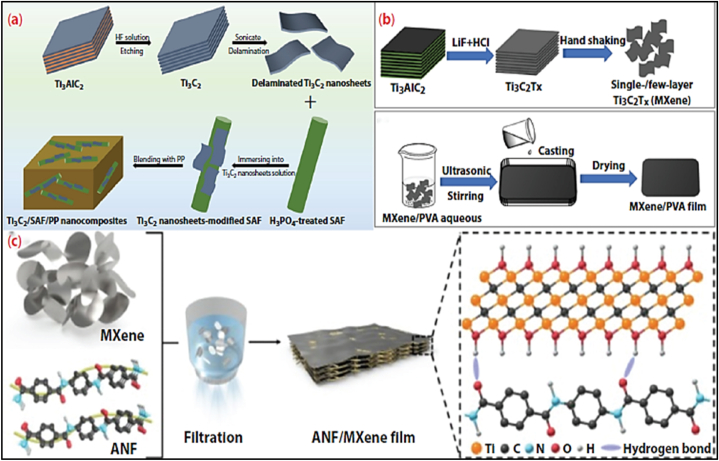
MXene-reinforced polymer composites are created by physically integrating MXene with polymer matrices. Ti3C2Tx/SAF/PP composites are synthesized through the melt-mixing technique. (a) Composite films of PVA/MXene are fabricated using the casting method. (b) PVA/MXene composite films are prepared using the casting process. (c) ANF/MXene films are produced via the filtration method.

**Source:** [135]**.**

An instance of this is the combination of polyvinyl alcohol (PVA) with MXene, which can be blended using sonication and stirring [130]. The resulting mixture may then be poured into molds and dried to produce uniformly disseminated composites (shown in Fig. 7). This technique, renowned for its straightforwardness and ability to be manipulated, is also employed in the production of composites, including polymers such as polystyrene (PS), poly(3,4-ethylenedioxythiophene) (PEDOT), and polyvinyl chloride (PVC) [131]. The process of solution suction filtration can be employed to manufacture polymer membranes reinforced with MXene. The procedure entails dissolving a polymer in a polar solvent, thoroughly mixing it with MXene through vigorous stirring and sonication, and filtering the solution to create a composite membrane [132]. MXene's high hydrophilicity enables its combination with aramid nanofibers (ANFs) to produce a multilayer film composite using vacuum-assisted filtering [133].

#### 3.1.2 Surface modification

Although the practice of combining MXene with polymers to produce MXene-reinforced polymer composites is widespread, various obstacles must be overcome [[136], [137]]. The hydrophilic nature of MXene restricts its compatibility with hydrophobic polymers including polydimethylsiloxane (PDMS), epoxy resin (EP), and polyvinylidene fluoride (PVDF) [138]. Surface modification techniques can alter the hydrophilicity or hydrophobicity of MXene, hence enhancing its compatibility with both hydrophilic and hydrophobic polymers. In addition, these treatments introduce chemical cross-linking sites that improve the stability and mechanical characteristics of the resultant composites. Surface changes can introduce functional groups such as -NH_2_, -SO_3_ and -NO_2,_ which can regulate the oxidation state of MXene and enhance its conductivity and capacitance [139]. An instance of this is the utilization of nitrogen-doped MXene nanosheets, which possess a well-defined porosity structure, as hosts for sulfur in lithium-sulfur batteries. Moreover, the formation of covalent bonding, such as the Ti-O-C link, can occur between MXene and other compounds via certain processes [140]. Functional groups such as carboxyl, amino, and long-chain alkanes can be used to chemically modify the hydroxyl groups on the surface of MXene. This modification aims to improve the interactions between MXene and polymers. Adding stearic acid to MXene increases its resistance to water, which in turn improves its ability to mix well and boosts its physical characteristics when combined with a polylactic acid (PLA) matrix. Functional alkoxysilanes have the ability to form strong chemical links called covalent Ti-O-Si bonds. These bonds serve as a connection between inorganic MXene materials and organic polymers. This procedure enhances the interactions with hydrophilic and hydrophobic polymers [141].

**Fig. 8:**
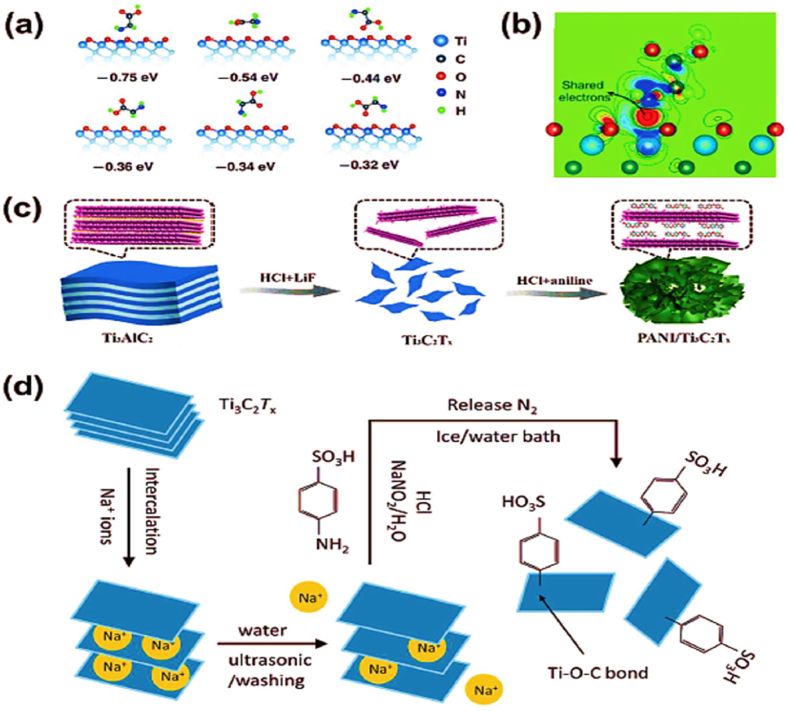
Composites made of polymers reinforced with MXene are created by altering its surface. (a) The N-MXene/S composites' preparation procedure. (b) HC-MXene/TiO_2_ electrodes are created mechanically, using Ti_3_C_2_Tx as a multipurpose conducting agent. (c) The PLA composites' preparation procedure. (d) EP preparation and PEPA-IPTS and PEPA-IPTS@ Ti_3_C_2_Tx synthesis.

Source: [143].

An instance is when Ti_3_C_2_T_x_ is combined with PEPA-IPTS to create nanohybrids. The surface groups of MXene (-OH, = -F, O) facilitate hydrogen bonding with polymers that possess functional groups such as carboxyl, hydroxyl and amine. As a result, the composites become stable and have a homogeneous distribution of MXene, which enhances their structural integrity in three dimensions. Yellow collagen gum and MXene can form film composites by forming hydrogen bonds(shown in Fig. 8). Moreover, negatively charged surface groups on MXene facilitate robust electrostatic interactions with positively charged polymers [142]. This allows for the creation of self-supporting MXene hydrogels that possess stable structures and exhibit outstanding mechanical properties, rendering them well-suited for use in biomedical applications.

#### 3.1.3 *In situ* polymerization

To improve the electrical conductivity and capacitance of MXene-reinforced polymer composites, the process comprises performing self-polymerization directly on the MXene surface, eliminating the requirement for initiators [[144], [145]]. This mechanism enables the transfer of electric charge between the polymer and MXene. During this transfer, electrons migrate between the highest occupied molecular orbitals (HOMOs) of MXene and the lowest unoccupied molecular orbitals (LUMOs) of the polymer, or vice versa. Monomers like 3,4-ethyl dioxythiophene (EDOT), aniline, and pyrrole can undergo polymerization on the MXene surface [146]. This process leads to an increase in the spacing between layers and the formation of conductive pathways. As a result, the electron-ion transport and the overall electrical characteristics of the composites are improved.

**Fig. 9:**
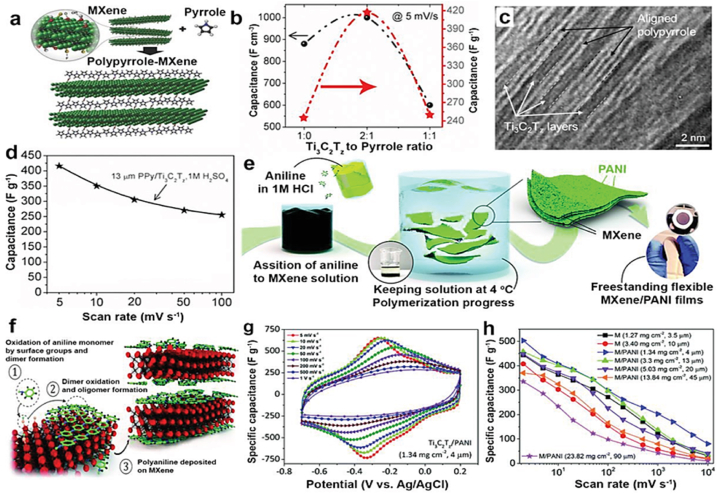
Polymers are deposited directly onto MXene surfaces using in situ methods. (a) Diagram illustrating the arrangement of PPy on the surface of MXene. (b) The impact of the pyrrole content on the volumetric and gravimetric capacitance of PPy/Ti_3_C_2_T_z_ hybrid electrodes is studied at a CV scan rate of 5 mV s⁻¹. (c) A cross-sectional transmission electron microscopy (TEM) picture showing the arrangement of PPy chains between MXene layers. (d) The ability of a 13 μm thick PPy/Ti_3_C_2_T_z_ electrode to handle high rates of operation. (e) Diagram illustrating the procedure used to produce the Ti_3_C_2_T_z_/PANI hybrid film. (f) Schematic illustration depicting the process of aniline polymerization on MXene surfaces. (g) Electrochemical voltage curves of the Ti_3_C_2_T_z_/PANI hybrid electrode at various scan speeds. Comparison of the rate capabilities of Ti_3_C_2_T_z_/PANI hybrid electrodes with varying mass loadings. M refers to electrodes made of pure Ti_3_C_2_T_z_, whereas M/PANI refers to electrodes that are a mix of Ti_3_C_2_T_z_ and PANI [150].

In situ polymerization entails the combination of polymer precursors, curing agents, and fillers in a solution, which is then subjected to polymerization under precise circumstances to produce macromolecules. The utilization of this technique is extensive in the preparation of polymer composites reinforced with MXene, such as polyurethane (PU), polyacrylamide (PAM), epoxy resin (EP), and polymethyl methacrylate (PMMA) [147]. When MXene is combined with EP, the EP molecule chains infiltrate the MXene, expanding the layer spacing and reinforcing the molecular structure during the curing process. An alternative and practical approach is using in situ electrochemical polymerization, particularly for conductive polymers. A two-step procedure was devised to synthesize Ti_3_C_2_T_x_/polypyrrole (PPy) hybrid membranes (Fig. 9). Initially, uniform membranes consisting of Ti_3_C_2_T_x_ particles are created through electrophoresis deposition. This is then followed by the in situ polymerization of pyrrole injected into the membranes [148]. This technique generates extremely thin, fully solid-state supercapacitors that exhibit exceptional cycle stability and capacitance. It presents a novel approach for creating MXene/conductive polymer membranes with remarkable electrochemical performance, which holds significant potential for energy storage applications [149].

#### 3.1.4 Hot press

MXenes demonstrate enhanced thermal stability and substantially elevated breakdown temperatures compared to polymers [[151], [152]]. During the hot-press production, MXene is mixed with a molten polymer matrix using a suitable high-powered mixer at a regulated temperature [153]. Subsequently, the mixture undergoes heat pressing to generate the ultimate nanocomposites. This approach offers several benefits, including being free of solvents, adaptable to various applications, cost-efficient, and environmentally friendly. As a result, it is well-suited for large-scale production. Hot-pressing methods have been employed to integrate MXenes into other polymers, including ultra-high-molecular-weight polyethylene (UHMWPE), linear low-density polyethylene (LLDPE), polyurethane (PU), polystyrene (PS), polyaniline (PANI), and polyvinylidene fluoride (PVDF) [154]. Contrary to solution-based approaches, which may have difficulty dispersing and dissolving MXene flakes, hot pressing is more efficient, particularly for hydrophobic polymers [155].

#### 3.1.5 Other method

Examples of applications include energy storage, electromagnetic shielding, and water purification. Melt blending is a prominent technique employed by Sheng et al. and Cao et al. to produce MXene/polymer nanocomposites using extruders and internal mixers. Researchers at Nanjing Tech University successfully created stable composites of PU and MXene using emulsion blending and ball milling techniques. Zhou et al. researched layered composites frequently employed in sensing, water purification, and electromagnetic protection. They specifically generated Ti_3_C_2_T_x_/PCL and MWCNT/PCL films for energy storage [156]. Cao et al. have created fiber composites coated with MXene, including cellulose nanofiber [157]. Mayerberger et al. documented the production of MXene and poly(acrylic acid) nanocomposite fibers using the electrospinning technique [158]. Various techniques enhance the compatibility between MXene sheets and polymeric matrix, developing composites with enhanced specific properties and wide-ranging potential applications [159].

### 3.2 MXene polymer composite property

Table 2 presents the essential characteristics of MXene polymer composites, including electrical conductivity, mechanical robustness, thermal stability, and other pertinent qualities. MXenes are a unique category of 2D materials that consist of transition metal carbides, nitrides, or carbonitrides. They are well-known for their exceptional mechanical strength and advantageous electrical conductivity. The addition of MXenes to polymers can improve their characteristics, making them appropriate for a wide range of uses, such as flexible electronics, energy storage, and electromagnetic interference shielding (see Table 3).

**Table 2:** Methods for synthesizing MXenes for diverse purposes.

MXene application	Preferred physical characteristics	Established synthetic methodologies	Alternative synthetic techniques
Storage and conversion of energy through catalysis Catalytic electrodes are used in electrochemical, photochemical, or photoelectrochemical reactions to facilitate the catalytic process. Energy storage devices such as batteries and supercapacitors	Excellent electrical conductivity Adjustable transmitter for targeted absorption of analyte Water-loving properties and ability to be processed in solution	HF etching Electrochemical etching Alkali etching Halogen etching Lewis’s acid molten salt etching	There are no MXene synthesis methods that have not been examined for energy storage and conversion applications.
Electromagnetic and electronic EMI protection, antennas, optoelectronics, photonics, bioelectronics (MXtrodes), and electronics Electronic instruments that you wear	Excellent electrical conductivity Expansive surface area Excellent structural integrity Hydrophilicity and solution processability are important factors to consider for MXtrodes	HF etching	Halogen etching Alkali etching
Sensors and the environment Chemical (liquid or gas) Adding trace amounts of heavy metals Membranes (for separating gases and liquids)	Excellent electrical conductivity Water affinity and capacity to dissolve in a solution Adjustable Targeting specific adsorption of the analyte	HF etching	Electrochemical etching Alkali etching Halogen etching
Biomedical Antimicrobial, antibacterial Biosensors, cancer theranostics Drug delivery Adsorption	Hydrophilicity and solution processability Tunable T*x* towards selective adsorption of analyte Large surface area	HF etching Electrochemical etching Alkali etching	Halogen etching
Mechanical tribology (hardness, sticking, and greasing) Composites used in construction	Unable to Tx to change the amount of grease or stiffness Controllable layer of MXene Not many problems Large surface area	HF etching	Alkali etching Halogen etching Lewis acid molten salt etching

**Table 3:** Property summary of MXene polymer composite [160,161]**.**

Property	MXene/Polymer Composite	Value/Range	Description
Electrical Conductivity	MXene/Polyvinyl Alcohol (PVA)	10^3 to 10^5 S/m	High conductivity due to MXene's metallic nature
Mechanical Strength	MXene/Polydimethylsiloxane (PDMS)	Tensile strength: 10–70 MPa	Enhanced strength with flexibility.
Thermal Stability	MXene/Epoxy Resin	Stable up to 300 °C	Improved thermal resistance compared to pure polymer.
Flexibility	MXene/Polyurethane (PU)	High flexibility	Retains flexibility with added electrical properties.
Young's Modulus	MXene/Polyvinyl Alcohol (PVA)	2–3 GPa	Increased stiffness.
Barrier Properties	MXene/Polyethylene Oxide (PEO)	Excellent oxygen and water barrier	Improved gas and moisture resistance
Electromagnetic Interference Shielding	MXene/Polyetherimide (PEI)	Shielding effectiveness: 40–60 dB	Effective EMI shielding due to MXene's conductive network
Thermal Conductivity	MXene/Polyamide (Nylon)	1–5 W/mK	Enhanced thermal conductivity for heat dissipation.
Dielectric Constant	MXene/Polyvinylidene Fluoride (PVDF)	10–50	Increased dielectric constant for capacitors.
Chemical Resistance	MXene/Polytetrafluoroethylene (PTFE)	High chemical resistance	MXene contributes to resistance against solvents and acids.
Hydrophilicity	MXene/Polysulfone (PSU)	High hydrophilicity	Improved water affinity for filtration applications
Photocatalytic Activity	MXene/Polymethyl Methacrylate (PMMA)	High photocatalytic activity	Effective for environmental applications.

#### 3.2.1 Electrical property

Polymer composites frequently have challenges in terms of inadequate electrical conductivity. However, integrating nanofillers, coatings, and other alterations can substantially enhance this issue. MXene is highly efficient in this aspect, as its incorporation into polymers can increase the distance between layers and facilitate the connection at the molecular level, hence improving the composites' conductivity, dielectric, and piezoelectric properties [162]. The surface terminations of MXene have significantly impacted its electrical characteristics, and MXene of excellent quality can demonstrate conductivities reaching up to 11,000 S cm^−1^. Yury's team also created a MAX phase with a more precise stoichiometry, leading to MXene exhibiting outstanding oxidation resistance and a conductivity of 6000 S cm^−1^ over six months [163]. MXene-polymer composites demonstrate exceptional electrical conductivity, principally attributed to the metallic properties of the MXene layers. The material's conductivity can vary between 10 and 10^5 S/m, depending on factors such as the amount of MXene present, the type of polymer used, and the structure of the composite.

The electrical properties of MXene are primarily influenced by the d-orbital electrons of the metal components on its surface and can be further improved by chemical treatment. Highly conductive MXene films were successfully produced hydrothermal, resulting in conductivities of up to 1652 S cm^−1^. The Seebeck coefficient was also greatly improved, suggesting possible uses in thermoelectric and semiconductor industries [164].

The PVDF/Co/MXene films, produced using a CO_2_-assisted foaming technique, exhibited a significant 4.4-fold enhancement in conductivity by using only eight wt% of MXene [165]. This resulted in the attainment of excellent electromagnetic wave absorption properties. Moreover, Ti_3_C_2_T_x_ MXene exhibited remarkable inherent current output and power density when subjected to tensile strain, rendering it well-suited for electromechanical transducers. Adding hyperbranched polysiloxane (HPSi)-modified Ti_3_C_2_T_x_ to PDMS substantially improved the dielectric constant at 100 Hz. This demonstrates the potential of these materials for use in many applications that require high-performance dielectrics.

#### 3.2.2 Thermal property

Practical uses depend on polymer composites' thermal stability, thermal expansion coefficient (TEC) and heat conductivity. Due to their covalent bond structure and heat transmission by phonons, MXene-reinforced polymer composites have these features. Phonon scattering often has a low mean free path because it must cross many crystal areas or anharmonic coupling motions. MXenes, two-dimensional materials, exhibit thermal conductivities that exceed most metals and rival graphene, suggesting they could improve polymer thermal characteristics [166]. MXene-polymer composites generally exhibit improved thermal stability compared to pure polymers. The breakdown temperature may exceed 300 °C, contingent upon the polymer matrix and the MXene loading.

Wu et al. conducted a study which revealed that pure CNF exhibited negligible thermal conductivity, but the inclusion of MXene greatly increased thermal conductivity. As an example, a paper made of laminated CNF/MXene with 60 % MXene by weight showed an in-plane thermal conductivity of 14.93 W m-1 K-1. The study emphasized the impact of morphology and the heat-conductive network on thermal conductivity [167]. Two-dimensional MXene produced a three-dimensional conductive network, increasing bonding lengths and hydrogen bonds, which improved thermal conductivity. Zero-dimensional MXene dispersed randomly and impeded heat conduction [168]. Yu et al. found that pure polystyrene (PS) showed high thermal weight loss with little residual carbon. POSS-MXene boosted PS composites' initial decomposition temperature (Ti), maximum decomposition temperature (Tmax), and residual char content, improving thermal stability [169]. By incorporating 2 wt% of POSS-MXene, the temperature at which the material starts to degrade (Ti) and the maximum temperature reached during degradation (Tmax) were raised to 404 °C and 432 °C, respectively. Additionally, the residual char content increased to 2.33 wt%, demonstrating the ability of POSS-MXene to effectively prevent the release of flammable volatile gases. Additional analyses of epoxy (EP) composites containing Ti_3_C_2_T_x_ showed a reduction in thermal expansion coefficient (TEC) from 46.3 ppm/K for pure EP to 40.7 ppm/K for EP-1.0M at a temperature of 40 °C. This decrease illustrates the capacity of MXene to reduce motion inside the EP matrix, improving the thermal resistance in EP composites. The results highlight the considerable capability of MXenes to improve the thermal properties of polymer composites, rendering them appropriate for advanced uses [170].

**Fig. 10:**
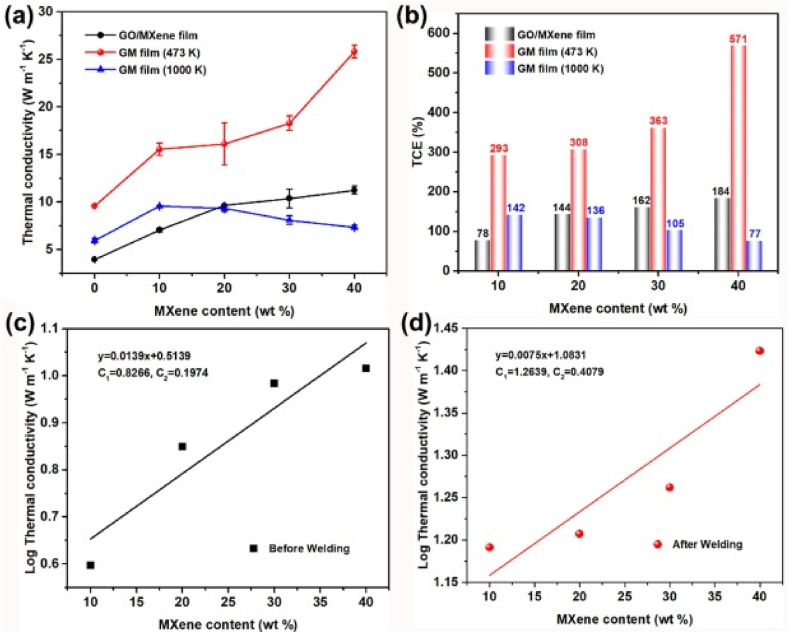
(a) Thermal conductivity (a) and Thermal conductivity enhancement factor (TCE) (b) of GO/MXene and GM film with different contents of MXen; Agari’s model fitting curve of the film (c) GO/MXene film, (d) GM film [171].

The epoxy/Ti₃C₂T_z_ nanocomposite exhibits enhanced thermal properties, including higher thermal conductivity, elevated glass transition temperatures, improved thermal stability, and a reduced coefficient of thermal expansion, as illustrated in Fig. 10.

#### 3.2.3 Mechanical properties

**Fig. 11:**
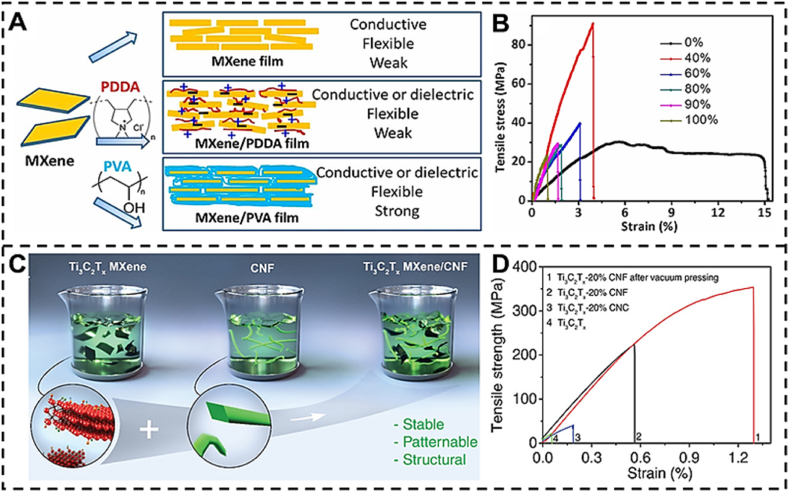
Mechanical characteristics of nanocomposite films based on MXene. (A) Schematic diagram showing the nanocomposite films based on MXene and pure MXene film. (B) The MXene-PVA nanocomposite films' stress-strain curves. Diagrammatic representation of MXene-CNF hybrid dispersion (C). (D) MXene-CNF nanocomposite film stress-strain curves both before and after vacuum pressing.

Reproduced from the study by Refs. [177].

Mechanical characteristics are key to polymer-based composites' widespread use. Nanofillers are often used to improve these qualities. S. Mishra found that adding 6 wt% nano-Mg(OH)_2_ to polypropylene (PP) enhanced tensile strength by 91 % and Young's modulus by 266 %. However, raising Mg(OH)_2_ to 12 wt% decreased tensile strength, emphasizing the importance of filler content. Research on MXene-reinforced polymers has grown, with MD, DFT, and FE simulations providing insights. Simulations predicted MXene's elastic modulus above 500 GPa, greater than most polymers. Young's moduli for Ti_3_C_2_O_2_ at 466 GPa and Ti_2_CO_2_ at 983 GPa exceeded those of pure graphene oxide (GO) in specific investigations [172]. Atomic defects like wrinkles and layers can lower mechanical characteristics. In practice, MXenes have been added to polymer composites in various ways. Polyurethane (PU) nanocomposites containing 0.5 wt% MXene improved tensile strength (20 %), hardness (13 %), and yield stress (70 %) utilizing an emulsion technique [173]. MXene was surface-treated with polyethylene glycol (PEG) to produce homogeneous dispersion in thermoplastic polyurethane (TPU), increasing storage modulus 39.8 % and tensile strength 47.1 %. Using sulfanilic acid-modified MXene as a reinforcing ingredient, Jeon Hyeon Woo created a new PVA film method. 0.5 wt% MXene and boric acid (BA) enhanced PVA film tensile strength by 67 % and modulus by 49 %. Another study found that adding 6.71 vol% MXene to natural rubber (NR) increased tensile strength and modulus 7- and 150-fold [174]. TPU with Zr-MXene had 43.4 % higher tensile strength than clean TPU. Adding 2 wt% Ti_3_C_2_T_x_ MXene to a polymer matrix increased impact strength by 50 % due to hydrogen bonding that improved interfacial contacts. These investigations show that MXene as a nanofiller can improve the mechanical properties of polymer composites for advanced applications [175,176]. Several physical characteristics of MXenes significantly impact the stress-strain response of epoxy/MXene nanocomposites (NCs). When the MXene nanosheets in these nanocomposites are oriented toward the applied load, the material may effectively resist deformation and provide the best reinforcement. Moreover, MXenes with large moduli give the composite strength and rigidity, improving its mechanical performance. MXene nanosheets with high aspect ratios improve reinforcing because their greater surface area allows for improved contact with the polymer matrix. Raising the volume fraction of MXene is advantageous when the intended mechanical properties for the nanocomposites are high moduli and tensile strength rather than elongation at break as illustrated in Fig. 11.

#### 3.2.4 Photo thermal properties

Light, a renewable and easily accessible kind of clean energy, may be efficiently harnessed to convert and treat heat through photothermal processes. MXene, renowned for its exceptional electrical conductivity, expansive specific surface area, minimal toxicity, outstanding biocompatibility, and elevated thermal conductivity, has emerged as a superb material for photothermal conversion. As a result, it has been utilised in several applications, including solar evaporation-driven actuators, biomedicine, and light heating systems [178]. A significant study by Feng et al. showcased a light heating system in which a self-floating MXene screen obtained an impressive 84 % efficiency in converting light into water evaporation under single sun irradiation. This efficiency is the greatest among several photothermal evaporation systems. The capacity of MXene to augment the heat storage capability of polymers suggests its potential for use in photothermal therapy.

Moreover, the ability of MXene to efficiently capture photon energy and selectively heat tumor cells while preserving the integrity of neighboring healthy cells has been well-documented. Nevertheless, the vulnerability of MXene to oxidation in aqueous and aerobic settings, which undermines its biological effectiveness, is a significant obstacle. Enhancing the biocompatibility of MXene while maintaining its photothermal efficiency can be achieved through polymer modification. Chen et al. found that when polyvinyl alcohol (PVA) is combined with molybdenum carbide (Mo_2_C), the resulting material exhibits excellent biocompatibility, fast degradation, and high photothermal conversion efficiencies of 24.5 % and 43.3 % for near-infrared (NIR)-I and NIR-II, respectively [178].

#### 3.2.5 Flame retardant properties

Two-dimensional lamellar nanoparticles like MXenes are good flame retardants due to their huge specific surface area and changeable composition. Catalytic metal composition reduces toxicity to release, while lamellar structure reduces mass and heat transfer during burning [179].

Zhu et al. found MXenes more flame-resistant than MAX phases. Compared to MAX, adding 2 wt% MXene to unsaturated polyester resin (UPR) composites reduced peak heat release rate (pHRR), total smoke production (TSP), and carbon monoxide production (COP) by 29.56 %, 25.6 %, and 31.58 %, respectively. Due to its wider layer spacing and surface area, MXene works well [179]. Liu et al. produced MXene at scale using wet ball milling and found that adding 3 wt% PDDA-MXene to thermoplastic polyurethane (TPU) lowered pHRR and TSP by 50 % and 47 %, respectively. The hybrid nanomaterial's synergistic effect on flame retardancy was shown by a 64.4 % pHRR reduction in CuP-MXene when 5 wt% was added to epoxy resin (EP) [180].

Organic methods like BODIPY modification prevented nanoparticle agglomeration. Yang et al. showed that BODIPY-treated MXene increased the limiting oxygen index (LOI) of acrylonitrile butadiene styrene (ABS) from 19.5 % to 23.5 % and decreased pHRR and PSPR by 24.5 % and 18.4 %, respectively. A UV-curable MXene composite coating on rigid polyurethane foam (RPUF) reduced heat and smoke and was self-extinguished. In conclusion, MXene and its hybrids greatly improve polymer composite flame retardancy, lowering fire dangers.

#### 3.2.6 Structural properties

The oxidation and rearrangement tendencies of MXene require adjustments to exploit its potential to greatly increase polymer matrices. Functional transformation enhances the dispersibility and compatibility of MXene, resulting in outstanding polymer composites. Tanvir et al. discovered that incorporating Ti_3_C_2_T_x_ MXene into polymer nanocomposites resulted in an enhanced storage modulus as the MXene content increased [181]. As confirmed by FT-IR analysis, Liu et al. successfully developed Mxene/alginate composites to effectively remove heavy metal ions from wastewater [182]. Liu et al. discovered that the addition of Ti_3_C_2_T_x_ enhanced the glass transition temperature and mechanical properties of Nafion [182]. This improvement was attributed to the formation of hydrogen bonds.

On the other hand, Zhang et al. investigated Ti_2_CT_x_ MXene-reinforced epoxy composites and observed substantial enhancements in fracture toughness, flexural strength, and impact strength. These improvements were attributed to the excellent interfacial compatibility between the components [183]. Raagulan et al. fabricated a nanocomposite coating by incorporating graphene oxide and MXene into a PVDF matrix, enhancing adaptability, wettability, and efficiency [184]. Wang et al. improved the fracture toughness of TPU composites by using Ti_3_C_2_ MXene, emphasizing the significance of PEG pre-treatment to achieve greater dispersion [185]. Sliozberg et al. employed MXene as an additive in a thermosetting polymer matrix to enhance its resistance to bending and cracking. This was substantiated by utilizing density functional theory (DFT) and molecular dynamics simulations. Liu et al. developed a PLA matrix reinforced with Ti_3_C_2_T_x_ MXene for bone regeneration, resulting in a notable 33 % enhancement in tensile strength. Similarly, Hu et al. enhanced the strength and resistance capabilities of Ti_3_C_2_ films by including chitosan [186]. Future research should prioritise the enhancement of the structural properties of MXene-based nanocomposites to better suit specific applications, making use of their outstanding capabilities in different scales and techniques.

#### 3.2.7 Tri-bological properties

Epoxy resins, a type of thermosetting polymers, are widely utilised in composite materials because they can easily incorporate reinforcements such as nanoparticles. MXenes, characterized by their stratified composition and presence of functional groups like hydroxyl, oxygen, and fluorine, have emerged as promising options for improving polymers' mechanical and tribological properties. Studies have demonstrated that including MXenes in epoxy resins greatly enhances their functionality in many applications, such as gears, bearings, and coatings [187]. Zhang et al. developed TiO_2_/Ti_3_C_2_ composite particles to improve the thermo-mechanical and tribological characteristics of epoxy resins. This resulted in reduced wear rates, thanks to the mechanical interlocking facilitated by TiO_2_ nanodots. Additional research, such as the tests by Zhang and colleagues on Ti_2_CT_x_ MXene/epoxy nanocomposites, have shown enhancements in toughness, micro-hardness, tensile strength, and flexural modulus. The strong contact between MXene nanosheets and the epoxy matrix, aided by functional groups, led to these improvements [188]. In addition, Meng et al. have created a new composite material called epoxy-Ti_3_C_2_ 3DNS, which has enhanced thermal and frictional characteristics [189]. In addition, Yan et al. conducted a study on the anti-wear properties of epoxy coatings that were improved using Ti_3_C_2_T_x_ sheets. They discovered that the wear rates and friction coefficients were significantly reduced by modifying the sheets and incorporating MXene, especially when combined with graphene [187]. The findings emphasize the capacity of MXenes to significantly enhance the longevity and efficiency of thermosetting polymer composites in diverse industrial applications [190].

#### 3.2.8 Interfacial strength

**Fig. 12:**
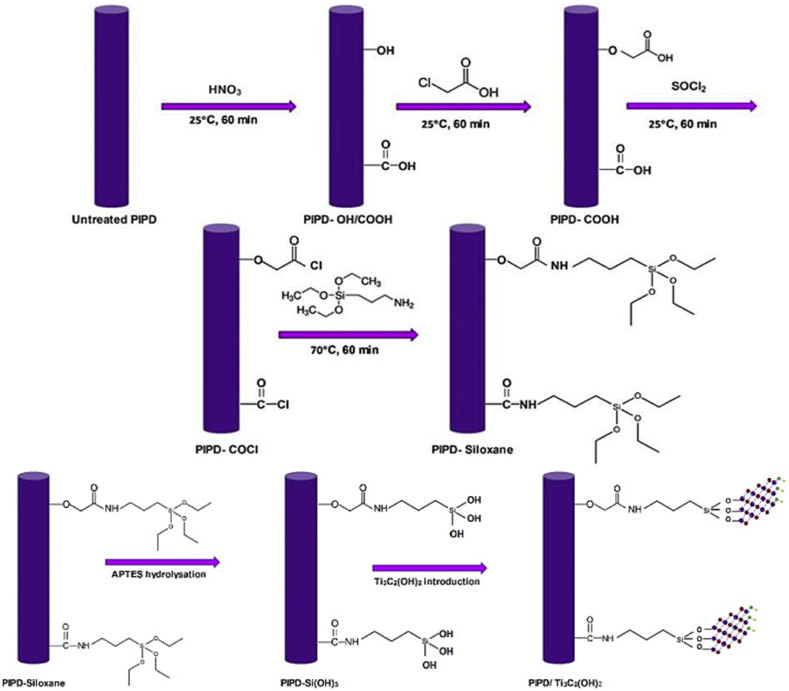
Schematics of silane functionalization of PIPD fibers and subsequent addition of Ti_3_C_2_(OH)_2_.

Reprinted from the study by [160].

The primary cause of the reinforcing effect in MXene nanocomposites (NCs) is the improved interfacial contact between the particles and the surrounding polymer matrix. Nevertheless, the definition of this relationship is typically ambiguous, and material qualities are commonly employed as markers of enhanced interfacial strength. Taloub et al. conducted a study to examine the impact of Ti_3_C_2_T_z_ grafting on PIPD fibers utilizing silane coupling. The results revealed a noteworthy 61.5 % enhancement in the interfacial shear strength (IFSS) when MXene was utilised. This improvement suggests improved contact and less particle aggregation [191]. This improvement is crucial even though the interface is directly between PIPD fibers and epoxy instead of the MXene and polymer matrix. The yield stress of nanocomposites (NCs) rises as the amount of filler, such as graphene and MXene, increases. This is because the interfacial contact is improved, leading to higher strength. This relationship may be mathematically described using a relative tensile yield stress equation. Generally, weak connections result in τ values below 10 MPa, whereas covalently coupled fillers demonstrate significantly larger values. Modeling of cross-linked PE/CNT nanocomposites suggests τ values ranging between 30 and 110 MPa [191]. Precisely quantifying and transparently disseminating the interfacial stress transfer parameter would significantly advance research on MXene polymer nanocomposites. In addition, Yu et al. created polyurethane (PU) nanocomposites by including MXene using an emulsification technique. They showed that adding 5 × 0.1 wt% MXene considerably increased the hardness, yield stress, and tensile strength by 10 %, 70 %, and 20 % correspondingly [192]. The improvement was ascribed to robust interfacial bonding, verified using FTIR analysis, underscoring the significance of even dispersion and efficient ball milling of MXene materials for enhancing mechanical strength and diminishing water absorption. A schematic of this surface functionalization is shown in Fig. 12. Although this is not a traditional NC, and the interface, in this case, is thought to be between the epoxy and the PIPD fibre rather than the strength of the interface between the polymer matrix and the MXene surface, the insights obtained indicate that coupling agents and surface functionalization can undoubtedly enhance the interfacial interactions and enhance dispersion by preventing particle agglomeration.

#### 3.2.9 AC electrical conductivity

The AC conductivity of composites is greatly improved by increasing the concentration of MXene from 0 to 4 wt percentage. At 25 Hz, the conductivity rises from 7 × 10^−11^ to 1.20 × 10^−5^ S m^−1^. A percolation threshold is seen at 4 wt%, beyond which there is a significant increase in electrical conductance. This occurrence is consistent with percolation theory, which suggests the creation of a network that allows for the flow of electricity and extra small capacitors within the material. This indicates an enhancement in the polarisation between PMMA and MXene at their interface [193].

In addition, ternary composites of MXene-ZnO nanoparticles demonstrate a slightly greater alternating current (AC) conductivity than binary composites. Specifically, composites consisting of PMMA/4:2 MXene: ZnO nanoparticles achieve an electrical conductance of 4.88 × 10^−5^ S m^−1^ at a frequency of 25 Hz. The increase in conductivity is attributed to the exceptional electrical conductivity of MXene and the partially conductive properties of ZnO NPs. These ZnO NPs serve as connectors, improving the composite's overall conductivity. A composite material of Ti_3_C_2_T_x_ MXene and PVDF was synthesized using several processes. This composite exhibited a significant dielectric constant of 41 and a low dielectric loss of 0.028 at a frequency of 1 kHz. Furthermore, it retained substantial dielectric characteristics even when subjected to high frequencies [194]. The complex structure hinders the creation of a pathway for electrical conduction, hence decreasing the loss of electrical energy and increasing the density of discharged energy. Feng et al. enhanced the dielectric constants and breakdown strengths of MXene composites by integrating PVDF/graphene, capitalizing on interlayer quantum confinement and Coulomb barrier effects. Quantum dots made of GO/N-doped Ti3C2-MXene, which can be found in binary and ternary composites, demonstrated improved dielectric characteristics due to the combined effects of synergy [195]. The AC conductivity of these composites exhibited substantial enhancements at the percolation threshold, aligning with the observed trends in dielectric loss. This suggests that the filler loading was efficient and resulted in improved electrical characteristics.

#### 3.2.10 Dielectric constant and dielectric loss

A recent study has emphasized the increased dielectric constant and interfacial polarisation in binary composites (PMMA/MXene and PMMA/ZnO) and ternary composites (PMMA/MXene/ZnO). In the case of binary composites, the dielectric constant experienced a substantial rise from 27.024 at a frequency of 25 Hz when 1 wt% MXene was added to 66.776 when the concentration of MXene was increased to 4 wt% [196]. The improvement is credited to developing nano-capacitors and interfacial polarisation, elucidated by the Debye model and percolation theory. The dielectric constant decreases as the frequency increases, ranging from 66.776 at 25 Hz to 36.276 at 1 MHz for a 4 wt% concentration of MXene in PMMA. Ternary composites demonstrate a significantly elevated dielectric constant of 435 at 25 Hz, suggesting greater surfaces and enhanced polarisation [197]. Adding MXene to polymers such as PMMA and PVDF greatly enhances their dielectric characteristics and conductivity.

An example is the enhancement of epoxy resin conductivity to 4.52 × 10^−4^ S cm^−1^ by using 0.85 wt% of Ti_3_C_2_T_x_ MXene. The dispersion of PVDF was improved by functionalizing MXene with CTAB, resulting in a dielectric permittivity of 82.1 and a low dielectric loss of 0.1 at a frequency of 1 kHz [198]. The study also found that when the frequency and MXene concentration increased, the dielectric loss decreased in binary and ternary composites. This indicates that these materials are well-suited for use in capacitors. As an illustration, the dielectric loss of a mixture containing 4 wt percent of MXene in PMMA was measured to be 0.04 at a frequency of 1 MHz [199]. In contrast, composites consisting of 4 wt percent MXene and 2 wt percent ZnO nanoparticles exhibited higher dielectric losses of 0.08 at the same frequency. The study demonstrates that the concentration of MXene and the inclusion of ZnO NPs substantially impact the dielectric characteristics and loss behavior, hence offering a wide range of potential applications in energy storage devices [200].

#### 3.2.11 Breakdown strength

The dielectric constant and breakdown strength are critical parameters that significantly affect the energy density of dielectric capacitors. One way to increase the ability to withstand breakdown is by combining semi-conductive ceramic particles with conductive MXene fillers in a polymer matrix, which helps to decrease leakage at the interface [201]. An example of a ternary composite consisting of polymer and V2C-CuO (10 wt%) demonstrated a significantly high breakdown strength of 204 MV m^−1^. Feng et al. examined the electrical characteristics of binary and ternary nanocomposites consisting of PVDF/graphene oxide (GO) and PVDF/GO@MXene. It was noted that the dielectric constant of binary PVDF/GO composites increased progressively when the filler reached 0.12 wt%. After this time, there was a sudden rise in the dielectric constant, which suggests the development of a conductive GO network. On the other hand, the ternary PVDF/GO@MXene composite did not exhibit a sudden rise, indicating that the impact of the percolation limit is less significant [202]. Similarly, the dielectric loss exhibited a sudden spike in binary composites but showed a steady variation in ternary composites. Both binary and ternary composites exhibited a decrease in electric breakdown strength as the filler content rose. However, the fall was less pronounced in ternary composites. Combining graphene oxide (GO) and nitrogen-doped titanium carbide (Ti_3_C_2_-MXene) quantum dots through hydrogen bonding self-assembly led to the formation of ternary composites. The composites exhibited higher dielectric constants and breakdown strengths compared to the binary counterparts, which can be attributed to the synergistic effects among their components [201]. The ternary nanocomposites demonstrated improved performance by including 0.12 wt% MXene, resulting in a dielectric constant of 53 and a breakdown strength of 205 MV/m at 1 kHz [203,87].

## 4 Novel applications of Polymer/MXene composites

MXenes, renowned for their exceptional characteristics, possess diverse applications across multiple domains. They are employed in energy storage, electromagnetic interference (EMI) shielding, microwave absorption, biosensors, water purification, and nanomedicine. MXenes possess many applications, such as pliable thin films, wearable materials, anchors in hybrid systems, or integrated into diverse matrices. Due to their improved electrical conductivity and capacity to enable ion diffusion, these electrodes show great potential for use in lithium, sodium, and aluminum-ion batteries. These batteries store charge by employing pseudocapacitive mechanisms that include surface redox reactions.

MXenes have proven highly efficient in environmental applications, particularly as absorbents and membranes for separating contaminants such as metal ions, gases, and dyes. In addition, they are utilised as gas sensors that can selectively capture carbon dioxide (CO_2_) and detect gases such as ethanol, acetone, ammonia, and methanol at normal room temperature. EMI shielding has attracted considerable attention among these applications because of the flexibility, ability to produce thin films, and excellent conductivity of MXenes. The wide range of applications demonstrates the extensive capabilities of MXenes in developing technology and tackling environmental issues.

### 4.1 Sensors

**Fig. 13:**
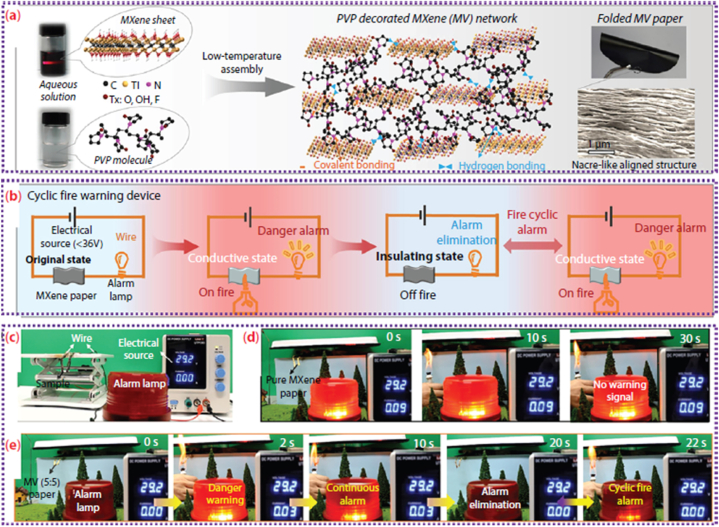
(a) Production illustration of MXene paper. (b) Modeling demonstration and (c) Digital photographs of the fire sensor. Fire sensor operation stages showing (d) Pure MXene paper and (e) Conductivity characteristics of PVP-modified MXenes.

Reprinted from the study by Ref. [207].

Recent research emphasizes the outstanding efficacy and durability of MXene-reinforced polymer sensors in a wide range of applications, such as pressure, humidity, temperature, and strain sensing. These sensors exhibit rapid development and performance enhancements comparable to the swift growth of bamboo shoots following rainfall. An example is a piezoelectric film created by electrospinning PVDF-TrFE with 20 wt% MXene. This film produced a consistent power density of 3.64 mW m^−2^ when subjected to a 2 N load at a frequency of 1 Hz [204]. As a result, it is well-suited for detecting human motion. In addition, a wearable fabric containing MXene on cotton demonstrated an exceptionally rapid response time of less than 35 ms and remained stable until 2000 cycles. An electrospinning and self-assembly technique created a 3D MXene framework capable of detecting volatile organic compounds (VOCs) at deficient parts-per-billion concentrations as illustrated in Fig. 13. The researchers utilised MXene and thermoplastic polyurethane to develop a fire-resistant paper that may provide early fire alerts, has excellent flame retardancy, and possesses form memory that can be activated by either heat or electricity. The films of Ti_3_C_2_T_x_ MXene modified with polyvinyl pyrrolidone exhibited remarkably rapid-fire warnings (1.8 s) and recovery durations (1.0 s) [205]. These impressive results can be attributed to the oxidation of MXene generated by thermal pyrolysis and the creation of a conductive titanium network. The MXene-based composites exhibit rapid-fire response, robust signals, and excellent cyclic stability, suggesting their suitability for advanced sensor applications in the future [206].

### 4.2 Energy sector

**Fig. 14:**
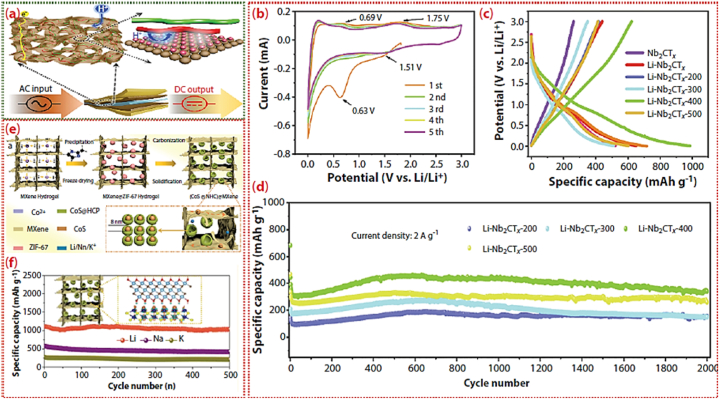
(a) MXene-based ECs. Source (b) Cyclic voltammetry (CV) of the Li-Nb2CTx-400 electrode (scanning rate: 0.5 mV s-1). (c) Galvanostatic discharge-charge (GDC) results of the initial cycle for different Nb2CTx electrodes at 0.05 A g-1. (d) Cycling performance in terms of specific capacity for samples annealed at 2.0 A g-1. (e) Production process of the MXene aerogel. (f) Capacity cycling performance of the aerogel.

MXenes have attracted considerable interest in the energy storage industry, specifically for their outstanding electrical characteristics, stability, and diverse surface groups. They are particularly promising for use in lithium batteries and supercapacitors. MXene/polymer electrochemical capacitors have exhibited remarkable performance, including a high areal capacitance of 0.56 mF cm^−2^ and a volumetric capacitance of 24.2 F cm^−3^ at 120 Hz. Additionally, these capacitors have shown long-term durability, enduring over 3000 cycles during bending testing [208]. The thickness and design ratio of Ti_3_C_2_Tx to PEDOT:PSS impacted the system's performance. The all-solid-state micro-supercapacitors fabricated using MXene ink exhibited an aerial capacitance of 1035 mF per square centimeter at a 2 mV per second scan rate. A three-dimensional FeNi-layered double hydroxide (FeNi-LDH) material was created using a two-dimensional Ti_3_C_2_Tx MXene [209]. This material exhibited a gravimetric capacitance of 922.6 F g^−1^ and maintained 88 % of its capacitance after undergoing 10,000 cycles. Polyaniline (PANI) composites containing 15 wt% MXene maintained 96 % of their capacitance after undergoing 10,000 cycles at a rate of 50 mV s^−1^, resulting in a capacitance of 434 F g^−1^ as illustrated in Fig. 14. This improvement can be attributed to the robust connection between PANI and MXene surface groups. A 3D porous MXene foam electrode was used to tackle the issue of MXene aggregation and restacking. This electrode exhibited a capacity of 455.5 mAh g^−1^ at a current density of 50 mA g^−1^ and an impressive rate capability of 101 mAh g^−1^ at 18 A g^−1^ [208]. In addition, the Li + -intercalated Nb_2_CT_x_, which was heated to 400 °C, attained a capacity of 448 mA-hours per gram (mAh g^−1^) at a rate of 0.05 A-hours per gram (Ah g^−1^) and maintained 75 % of its capacity after 2000 cycles at a rate of 2 A-hours per gram (A g^−1^). The aerogel structure of MOF@MXene, filled with hollow CoS, exhibited exceptional performance. It achieved capacities of 1145 mAh g^−1^ after 800 cycles at 1 A g^−1^ and 574.1 mAh g^−1^ after 1000 cycles at 5 A g^−1^ [210]. The results emphasize the possibility of producing MXene-reinforced polymers on a wide scale. However, there are still obstacles to overcome, such as aggregation, limited storage duration, and the need for quality control measures [211].

Source [135].

### 4.3 Electromagnetic interference shielding

**Fig. 15:**
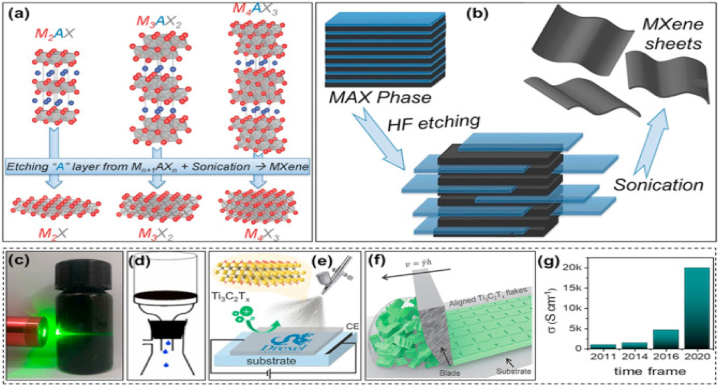
(a)The atomic structure of various MAX phases can be determined by considering the number of ‘M' layers and the matching 2D MXenes obtained by removing the ‘A' layer through etching. (b) Diagram showing the process of creating MXene sheets from compressed MAX phases. (c) An aqueous dispersion of Ti_3_C_2_T_x_ MXene exhibits the Tyndall effect and can be processed into various shapes using (d) vacuum-assisted filtering, (e) spray coating, and (f) blade coating. (g) The electrical conductivity of MXenes, particularly Ti_3_C_2_T_x_, normally improves over time.

In the era of 5G technology, it is essential to design electronic equipment that is small and lightweight. Additionally, there is a growing urgency to reduce the amount of electromagnetic (EM) wave radiation. Despite being lightweight and cost-effective, conventional polymers frequently do not possess electromagnetic wave resistance. MXene-reinforced polymers are being recognized as an auspicious approach for creating conductive devices that can be adjusted to have the desired electromagnetic interference (EMI) performance [212]. In 2016, Professor Cheng and colleagues published a study stating that a 45 μm Ti_3_C_2_T_x_ film demonstrated an electromagnetic interference (EMI) shielding efficiency (SE) of up to 92 dB, establishing a notable standard [213]. Subsequently, many inventive methods have improved composites' electromagnetic interference (EMI) characteristics based on MXene, as shown in Fig. 15 [214]. Electrical conductivity (σ) is of utmost importance in electromagnetic interference (EMI) shielding. A minimum conductivity of 0.1 S/mm is necessary for shielding applications to be effective. The MXene film demonstrates a notable electrical conductivity of 247.7 S/mm, contributing to its exceptional electromagnetic interference (EMI) shielding capabilities. Nevertheless, the conductivity of MXene/NR composite films diminishes as the proportion of natural rubber (NR) increases, ranging from 145.1 S/mm for MXene/NR10 % to 12.1 S/mm for MXene/NR60 %. Although there has been a decrease, the conductivity of MXene/NR60 % still above the minimum threshold of 0.1 S/mm required for EMI applications [215].

Source [161].

An example of this is the production of flexible foam with an electromagnetic interference shielding effectiveness (EMI SE) of up to 70 dB by the use of hydrazine-induced foaming of MXene. This foam is well-suited for applications in aerospace and smart devices. By combining Ti_3_C_2_T_x_ with polyvinyl alcohol (PVA), a porous composite material was formed that exhibited an outstanding SSE/t value of 5136 dB cm^2^g^−1^. Additional progress has been made by incorporating MXene-decorated polystyrene (PS) beads into a polydimethylsiloxane (PDMS) enclosure, resulting in electromagnetic interference shielding effectiveness (EMI SE) values ranging from 23.5 to 39.8 dB in the X band [216]. Attempts to develop environmentally friendly and biodegradable electromagnetic interference (EMI) composites have produced delignified wood and d-MXene composites. The MXene/NR composite films have an EMI shielding effectiveness (SE) that surpasses 30 dB throughout the X-band, regardless of the concentration of NR (10, 20, 40, or 60 wt%). This exceeds the commercial requirement of 20 dB. The observed shielding efficiency reaches a maximum value of over 99.996 %, which indicates an outstanding level of electromagnetic interference (EMI) shielding effectiveness. The specific absorption ratio (SSE) of MXene/NR composites, which indicates the efficacy of shielding in relation to thickness, is remarkably high, particularly for MXene/NR40 % and MXene/NR60 %. This demonstrates that even with extremely thin layers, these composites provide effective electromagnetic interference (EMI) shielding [217].

These composites have an EMI shielding effectiveness (SE) of 39.3 dB at a frequency of 12.4 GHz. Solution casting was used to create annealed MXene/epoxy composites and sandwich-structured films. These materials were enhanced by introducing Ca^2+^-crosslinked sodium alginate and montmorillonite (MMT). The resulting composites and films demonstrated improved electromagnetic interference shielding effectiveness (EMI SE) values of 41 dB and 50.01 dB, respectively [218]. In addition, when subjected to strain, sheets made of thermoplastic polyurethane (TPU) covered with stretchy MXene exhibited an electromagnetic interference shielding effectiveness (EMI SE) of 30 dB. The various techniques showcase the adaptability and exceptional effectiveness of polymers reinforced with MXene. In addition, the ability of MXene/NR60 % films to shield against electromagnetic interference (EMI) increases as the thickness of the film increases. The shielding efficacy ranges from 28.5 dB for a 12.3 μm film to 54.2 dB for a 131 μm film. The minimum thickness needed to meet the commercial criterion of 20 dB is less than 12.3 μm. The MXene/NR60 % film, with a thickness of 131 μm, exhibits an impressive shielding efficiency of 99.9996 % and a transmission rate of only 0.0004 %, showcasing its exceptional performance as an electromagnetic interference (EMI) shielding material [219].

Nevertheless, obstacles such as irregular lamella diameters, problems with dispensability, and vulnerability to oxidation continue to hinder their extensive use. Notwithstanding these difficulties, the progress made in MXene-based EMI composites highlights their promise in future electronic devices.

### 4.4 Catalytic conversion

Catalysts play a crucial role in the global economy by supporting more than 90 % of chemical reactions necessary for industrial operations, especially during energy and oil crises. MXenes, a type of 2D material, are considered extremely reactive catalysts because they feature many hydrophilic groups on their surface, including = O, -F, and -OH [220]. These groups can be modified to customize MXene's selectivity for specific reactions. MXenes exhibit metallic conductivity, promoting effective electron and proton transport and improving their catalytic efficiency. MXenes have been utilised in various fields, such as transforming harmful flue gases, purifying contaminated wastewater, converting carbon dioxide, and photothermal catalysis. These applications showcase the extensive capabilities and substantial potential of MXene in tackling current environmental and industrial concerns [221].

### 4.5 Oil/water separation

Crude oil leaks and oil pollution are major environmental concerns, and recent advancements in treatment techniques have prioritized eco-friendly and energy-efficient measures. MXene-based materials have demonstrated significant potential in this field due to their distinctive characteristics and architectures [222]. In 2019, Zeng experimented to show that a polyimide aerogel, created by combining polyimide with MXene and then freeze-drying it, displayed a remarkable ability to separate oil and water [223]. The aerogel absorbed 18–58 times its weight in different organic solvents. In addition, the integration of halloysite nanotubes and polydopamine on MXene surfaces yielded a material that exhibited a pure water flux of 5036.2 L m^−2^ bar^−1^ and a rejection rate of 99.8 % for petroleum ether and lubricating oil [224]. In addition, membranes made of polyacrylonitrile (PAN) modified with MXene, as well as membranes coated with MXene/β-FeOOH on polyvinylidene fluoride (PVDF), had outstanding performance in separating oil and water [225]. These membranes demonstrated high water permeability and separation efficiencies over 99 % [226]. A conductive polyimide/MXene aerogel, produced through freeze-drying, exhibited significant absorption capabilities ranging from 55.85 to 135.29 g g^−1^. The results highlight the ability of MXene-based polymers to efficiently separate oil and water, making them appealing for use in industrial settings [227].

### 4.6 Detection of VOCs and hazardous gases

MXenes have demonstrated outstanding performance in sensor applications due to their significant specific surface area, superior conductivity, and compatibility with living organisms. MXenes, specifically Ti_3_C_2_T_x_, are highly efficient in immobilising active proteins in biosensors [228]. They effectively preserve the bioactivity of these proteins and facilitate quick electron transfer between enzymes and electrodes. As a result, these biosensors exhibit exceptional sensitivity and selectivity without needing mediators. An instance of a biosensor employing Ti_3_C_2_T_x_ to fixate haemoglobin exhibited exceptional nitrite detection capabilities, with a minimum detectable concentration of 0.12 μM. MXenes effectively resolve the selectivity challenges encountered by traditional gas sensors [229]. Computational research has demonstrated that Ti_2_CO_2_ exhibits preferential adsorption of NH_3_ compared to other gases, resulting in the development of susceptible NH_3_ sensors. Gas sensors based on Ti_3_C_2_T_x_ can detect various gases, such as methanol, ethanol, NH_3_, and acetone. Among these gases, NH3 elicits the most pronounced reaction [230]. The sensing process entails gas absorption and desorption by functional groups on Ti_3_C_2_T_x_. Researchers have created MXene-based volatile organic compound (VOC) sensors that exhibit low power consumption and great selectivity [231]. These sensors are designed for monitoring purposes. The Ti_3_C_2_T_x_-TiO_2_ nanocomposites, produced using a hydrothermal technique, exhibit considerably enhanced sensitivity to volatile organic compounds (VOCs) compared to pure MXene. The gas response to 10 parts per million (ppm) of hexanal is around 3.4 %. In addition, the combination of MXenes and two-dimensional materials such as WSe_2_ has led to the development of sensors that exhibit rapid response times, swift recovery, little noise, and versatility in detecting different volatile organic compounds (VOCs). The detecting process is based on variations in conductivity caused by electron transfer between the nanohybrids and gases that are adsorbed, which improves the sensor's ability to detect and distinguish different gases. The developments underscore the potential of MXenes in the development of exceptionally effective biosensors and gas sensors [232].

### 4.7 Engineering biosensors and bioimaging platforms

MXene composites are widely used as adaptable platforms for biosensors and imaging applications because they can easily be modified and functionalized on their surface. MXenes possess a negative surface charge due to functional groups such as hydroxyl, oxy, and fluoride. This property allows them to attract and bind positively charged particles through electrostatic contact selectively. Consequently, MXenes can be utilised to create biosensors that can detect and analyze various substances in biomedical applications [233]. For example, TiO_2_- Ti_3_C_2_ MXene and Nafion nanocomposites have created biosensors without a mediator to detect hydrogen peroxide. These biosensors have shown great compatibility with proteins for immobilisation and have improved the transmission of electrons in detecting haemoglobin (Hb). MXenes have also been utilised in biosensors called organic electrochemical transistors (MOECTs) to detect prostate-specific antigens (fPSA/tPSA) [234]. These biosensors have shown excellent sensitivity and can detect very low levels of antigens. In addition, Ti_3_C_2_T_x_ MXene nanosheets have been employed in glucose biosensors, providing highly sensitive detection abilities and showing potential uses in diagnosing Diabetes Mellitus. Moreover, MXenes have been utilised in point-of-care immunoassay biosensors to detect viruses such as H1N1 and cholesterol biosensors and taste-sensing applications in the food industry [235]. This demonstrates their adaptability and potential in several biological fields. In addition, MXenes have been integrated into innovative biosensors designed to detect cancer biomarkers like carcinoembryonic antigen (CEA). These biosensors provide exceptional sensitivity, specificity, and accuracy for clinical diagnostics. In summary, the distinctive characteristics of MXenes make them highly favorable options for creating cutting-edge biosensors and imaging systems with a broad range of biomedical uses, including illness detection and treatment tracking [236].

### 4.8 Drug delivery system and photothermal therapy

**Fig. 16:**
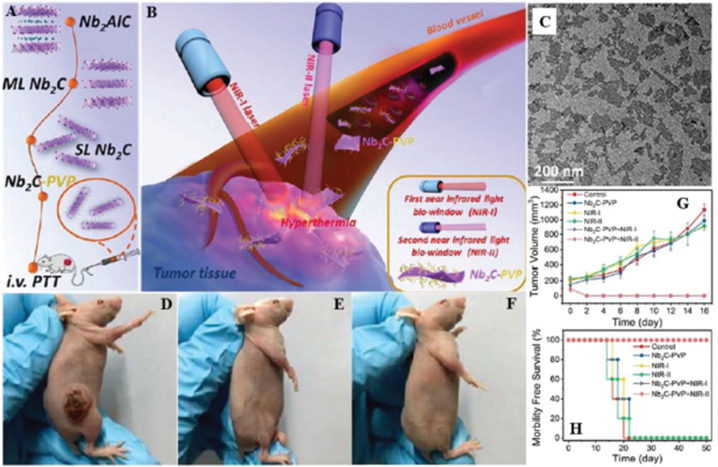
Diagrammatic representation of PVP-modified 2D biodegradable Nb2C for in vivo photothermal tumor ablation in NIR-I and NIR-II bio-windows (A) and (B). TEM picture of Nb2C-PVP/PBS (C). Photographs (D–F) showing the tumor regions and 4T1-bearing mice 16 days following various treatments (control (D), Nb2C-PVP + NIR-I (E), and Nb2C-PVP + NIR-II (F)). (G) Tumor growth curves that rely on time (n = 5, mean ± SD) following various treatments. (H) Mice survival curves following different treatments.

One of the biggest health risks today is cancer, which necessitates novel approaches to treatment in addition to traditional methods like radiation, chemotherapy, and surgery, which frequently fall short of fully eliminating cancer cells and can have serious adverse consequences by destroying good tissues. Due to the low heat resistance of cancer cells, photothermal treatment (PTT), which converts near-infrared (NIR) light into heat, has drawn interest as a potential solution to these problems. PTT mostly works in the first (750–1000 nm) and second (1000–1350 nm) NIR bio-windows. The latter has advantages such as deeper tissue penetration and higher allowable exposure, but its use has been restricted because there aren't many efficient photothermal agents that absorb in this range. Due to their effective photothermal conversion capabilities, MXene-based materials, namely titanium carbide (TiC₂) and niobium carbide (NbC₂), have recently demonstrated high potential for PTT throughout both NIR windows. Research findings indicate that Nb₂C coupled with poly(vinylpyrrolidone) (PVP) can efficiently eradicate tumors in mice, underscoring the potential of materials based on MXene to accomplish total tumor regression and prolong survival without recurrence [237]. MXenes are being investigated as chemotherapeutic carriers in addition to PTT, providing regulated drug delivery methods as illustrated in Fig. 16. Their effective accumulation at tumor locations is facilitated by their nanoscale size, and their enormous specific surface area permits substantial drug loading. Combining MXenes with anti-cancer medications such as doxorubicin (Dox) can maximize therapeutic impact while reducing negative effects since MXenes exhibit pH-responsive and NIR-accelerated drug release. Preclinical research has shown that the combined use of PTT and chemotherapy using MXene carriers produces better results, with a notable decrease in tumor size and little harm to healthy tissues. Under NIR irradiation, for example, TiC_C nanosheets treated with soybean phospholipid demonstrated great drug-loading capacity and effective transport to cancer cells, resulting in considerable tumor eradication [238]. Despite these developments, drug loading in MXene-based drug delivery systems frequently depends on non-covalent interactions, which may not always result in stable and regulated release. In order to get around this restriction and improve the dependability and effectiveness of these systems, scientists are looking into covalent and dynamic bonding techniques. The combination of the photothermal capabilities of MXenes with the drug-loading capacity of porous materials has led to innovations such as surface-nanopore engineering with mesoporous silica coatings, which enable precise control over drug release driven by pH changes and NIR light [239]. MXenes are potential materials for cutting-edge cancer therapy because of their special qualities, which include their vast surface area, high photothermal conversion efficiency, and simplicity of surface modification. They enhance biocompatibility, enable targeted and regulated drug release, and present an opportunity to combine various therapeutic methods. Upcoming studies seek to improve MXene-based systems by maximizing therapeutic efficacy and minimizing off-target effects by optimizing drug release profiles and adding active targeting mechanisms. The field of cancer treatment is progressing with the creation of MXene-based nanomaterials, providing promise for safer and more effective medicines in the battle against this tough disease [240].

Source [241].

## 5 Limitations and challenges

The advancement and utilization of MXenes pose several difficulties, specifically concerning their durability, expandability, and incorporation into diverse technologies. An important obstacle is the vulnerability of MXenes to oxidation, which impacts their effectiveness in applications that demand long-term stability, such as energy storage and sensing. It is imperative to tackle the problem of oxidation in order to ensure the practical application of these materials, particularly in environments that are humid or severe. Another major obstacle is the scalability of MXene synthesis. Existing techniques, frequently employing perilous substances such as hydrofluoric acid (HF), should be substituted with safer and more economically efficient alternatives. The scalability of MXene/polymer nanocomposites production is hindered by the challenge of preserving their distinctive features on a significant scale. The commercial applications of MXenes are hindered by the limited availability and high cost of MAX phase powders, which serve as precursors. It is crucial to undertake the development of efficient synthesis methods that do not depend on hydrofluoric acid (HF) and to optimise the production process in order to make it economically feasible for large-scale applications. Increasing the surface area of MXenes to be comparable to that of graphene has the potential to greatly enhance their energy density and specific capacitance in energy storage. Novel techniques, including acid and basic treatments, show promise in creating pores and enhancing surface area. However, additional research and refinement are necessary.

Thorough analysis is required to assess the biocompatibility, cell uptake behavior, and environmental toxicity of MXenes for biomedical applications. It is crucial to prioritise the safety of MXenes in therapeutic settings, which necessitates the establishment of systematic protocols and risk evaluations. MXene-based membranes exhibit promise in water purification and desalination, although their use is currently confined to experimental experiments conducted in laboratory settings. A thorough investigation is required to assess their potential for profitability and enhance their effectiveness in practical situations. Wearable sensor applications encounter difficulties due to the hydrophilic nature of MXenes, which might result in device deterioration under moist conditions. Using polymer reinforcement can alleviate these problems, improving the mechanical characteristics and stability of sensors based on MXene. Further investigation should prioritise comprehending the correlation between the manufacturing procedure and the physicochemical characteristics of MXenes. Improving the etchants to be less aggressive and increasing the speed of the production process are crucial areas for enhancement. In general, the future outlook for MXenes is highly favorable, as they hold great potential for use in several domains such as energy storage, biomedical applications, water purification, and wearable technology. Tackling the present obstacles by engaging in cutting-edge research and development will create the conditions for their extensive acceptance.

## 6 Future outlook

The prospects for MXene-based materials are highly promising, as they possess extraordinary qualities and continuing research is focused on addressing current obstacles. This bodes well for their prospective applications in various industries. MXenes are expected to play a crucial role in the development of high-performance batteries and supercapacitors in the field of energy storage. Although there are still challenges, such as the arrangement of MXene layers that hinders ion intercalation and decreases electrochemical performance, researchers are investigating inventive approaches such as structuring MXenes and creating hybrid materials based on MXene. These methods strive to boost the ability of electricity to flow, the durability of the materials, and the transfer of ions and electrons. This ultimately results in energy storage devices that have better performance in terms of charging and discharging quickly, as well as having a higher capacity to store energy. Future research will likely prioritise the quest for the best balance between high-rate performance and volumetric efficiency. Additionally, there will be an emphasis on comprehending and controlling surface terminations to adjust MXene characteristics precisely.

MXene-polymer nanocomposites are gaining recognition as an up-and-coming field for multifunctional applications alongside their energy storage capabilities. These composites merge the distinct characteristics of MXenes with polymers to produce materials that are both mechanically strong and exhibit improved electrical conductivity, thermal stability, and chemical reactivity. These nanocomposites are highly adaptable and can be effectively utilised in various fields, such as sophisticated electronics, sensors, biomedical devices, environmental remediation, and smart materials. Nevertheless, it is imperative to tackle scalability, repeatability, and stability difficulties. Subsequent studies will concentrate on the advancement of sustainable and eco-friendly techniques for synthesizing MXene, with an emphasis on minimizing the utilization of dangerous chemicals and mitigating the environmental consequences of its manufacture. To achieve these aims while maintaining environmental sustainability, it is crucial to employ green synthesis methods, energy-efficient production procedures, and recycling strategies for MXenes.

Furthermore, incorporating MXenes into additive manufacturing techniques such as 3D printing offers intriguing prospects for fabricating intricate structures with customised characteristics. This innovation has the potential to completely transform various industries by facilitating the quick creation of prototypes, personalised products, and the production of MXene-based items as needed. To fully harness the promise of MXenes in 3D printing, addressing the obstacles posed by material constraints, surface quality, and printing speed is crucial. MXene-polymer nanocomposites exhibit potential in the biomedical domain for several applications, including drug delivery systems, tissue engineering scaffolds, biosensors, and medical implants. Due to their biocompatibility, bioactivity, and capacity to provide controlled release features, they are highly suitable for future biomedical devices and therapies. With the ongoing advancement of personalised medicine, MXenes have the potential to be highly influential in the progress of targeted medicines, implantable devices, and improved diagnostic tools.

MXenes are anticipated to have a substantial influence in the field of environmental remediation. Due to their significant adsorption capacity, catalytic activity, and selectivity towards contaminants, they are well-suited for water purification, air filtration, soil remediation, and wastewater treatment. The advancement of MXene-polymer nanocomposites with intelligent and reactive properties has the potential to offer creative solutions for environmental issues.

Computational modeling and simulation approaches will be essential for the future advancement of MXene-based materials. These methods will facilitate the logical development and enhancement of MXene-polymer nanocomposites, enabling researchers to forecast material attributes, comprehend structure-property connections, and optimise performance in different circumstances. This will expedite the advancement of novel MXene-based materials and their incorporation into real-world applications.

In summary, MXene-based materials have many uses and the potential to revolutionise various sectors. As research progresses to tackle existing difficulties and investigate unexplored areas, MXenes are positioned to become a fundamental element of technological innovation, propelling improvements in energy storage, environmental preservation, biomedical applications, and more.

## 7 Conclusions

MXenes are a highly adaptable group of two-dimensional materials with remarkable promise in various applications owing to their distinctive characteristics, including exceptional conductivity, expansive surface area, and convenient functionalization. Nevertheless, certain obstacles must be overcome to exploit their capabilities thoroughly. Challenges related to oxidation and stability provide significant obstacles, especially for applications that demand sustained performance over extended periods. It is essential to employ inventive methods to improve the ability of MXenes to resist oxidation. The expansion of MXene production is another crucial area that requires attention. It is necessary to find safer and more economically efficient alternatives to the current technologies that use dangerous chemicals such as HF to make large-scale production feasible. Enhancing the surface area of MXenes in energy storage can significantly improve energy density and specific capacitance, enabling them to rival graphene.

Similarly, in water purification, the membranes made from MXene require additional progress and assessment in real-world scenarios to go from small-scale laboratory experiments to practical use in commercial settings. Extensive study is necessary to guarantee the biocompatibility and safety of MXenes for biomedical applications. Wearable sensors with MXenes demonstrate promise but encounter difficulties associated with their hydrophilic nature and stability in severe environments. Utilizing polymer reinforcement can improve the mechanical characteristics of materials and prolong their durability. Subsequent investigations should prioritise the enhancement of the synthesis procedures, elucidation of the correlation between the fabrication of MXenes and their properties, and the exploration of novel applications. To fully realise the potential of MXenes and encourage their wider use in different industries, it is crucial to tackle these problems by conducting focused research and developing new solutions. Ultimately, the successful incorporation of MXenes into commercial and industrial applications is contingent upon effectively addressing the existing obstacles despite the significant benefits they present. Through ongoing study and development, MXenes have the potential to become a fundamental material in future technologies.

## Data availability statement

No data was used for the research described in the article.

## CRediT authorship contribution statement

**Md Injamamul Haque Protyai:** Writing – original draft, Conceptualization. **Adib Bin Rashid:** Writing – review & editing, Methodology, Conceptualization.

## Declaration of competing interest

The authors declare that they have no known competing financial interests or personal relationships that could have appeared to influence the work reported in this paper.
